# Assessment of clinical analytical sensitivity and specificity of next-generation sequencing for detection of simple and complex mutations

**DOI:** 10.1186/1471-2156-14-6

**Published:** 2013-02-19

**Authors:** Ephrem LH Chin, Cristina da Silva, Madhuri Hegde

**Affiliations:** 1 Department of Human Genetics, Emory University, Michael Street, Atlanta, GA, USA

**Keywords:** Targeted, Enrichment, Next-generation, Sequencing, Mutation, Detection

## Abstract

**Background:**

Detecting mutations in disease genes by full gene sequence analysis is common in clinical diagnostic laboratories. Sanger dideoxy terminator sequencing allows for rapid development and implementation of sequencing assays in the clinical laboratory, but it has limited throughput, and due to cost constraints, only allows analysis of one or at most a few genes in a patient. Next-generation sequencing (NGS), on the other hand, has evolved rapidly, although to date it has mainly been used for large-scale genome sequencing projects and is beginning to be used in the clinical diagnostic testing. One advantage of NGS is that many genes can be analyzed easily at the same time, allowing for mutation detection when there are many possible causative genes for a specific phenotype. In addition, regions of a gene typically not tested for mutations, like deep intronic and promoter mutations, can also be detected.

**Results:**

Here we use 20 previously characterized Sanger-sequenced positive controls in disease-causing genes to demonstrate the utility of NGS in a clinical setting using standard PCR based amplification to assess the analytical sensitivity and specificity of the technology for detecting all previously characterized changes (mutations and benign SNPs). The positive controls chosen for validation range from simple substitution mutations to complex deletion and insertion mutations occurring in autosomal dominant and recessive disorders. The NGS data was 100% concordant with the Sanger sequencing data identifying all 119 previously identified changes in the 20 samples.

**Conclusions:**

We have demonstrated that NGS technology is ready to be deployed in clinical laboratories. However, NGS and associated technologies are evolving, and clinical laboratories will need to invest significantly in staff and infrastructure to build the necessary foundation for success.

## Background

The introduction of next-generation sequencing (NGS) has revolutionized the way sequencing is being conducted in many research and clinical laboratories. Large genome centers have been the early adopters of NGS and use it primarily for large-scale genome sequencing projects [1-3]. A single next-generation instrument is able to sequence a whole human genome at 7.4-fold coverage in two months [2]. In comparison, the International Human Genome Sequencing Consortium of 20 laboratories worldwide took approximately 15 months to perform the same work [4]. There are currently four major manufacturers of next-generation instruments, and they all share the same fundamental process using four different chemistries [5]. Third-generation sequencers, like the Ion Torrent and Pacific Biosciences systems, have emerged as viable alternatives to the four next-generation sequencers and have started to appear in laboratories [6,7].

In the last few years, clinical laboratories have begun to investigate how best to use the prodigious data-generation capacity of the NGS for clinical testing, as this tremendous sequencing capacity opens up new diagnosis possibilities that Sanger sequencing technology could not offer. Automated dideoxy Sanger sequencing has been the workhorse in clinical laboratories for many years and is considered to be the “gold standard” [8]. Clinical sequencing assays using Sanger sequencing are easy to develop and can be deployed rapidly in a clinical laboratory; however, it has limited data-generation capacity, mainly due to cost constraints, and it only allows analysis of one or at most a few genes in a patient. Accurate and sensitive mutation identification are of paramount importance for diagnosis confirmation, genetic counseling, risk assessment, and carrier screening in patients and family affected with a genetic disorder. The ability of a single next-generation sequencer to generate massive amounts of data allows a laboratory the opportunity to analyze many more genes in a cost-effective manner [9]. Many possible candidate genes for a specific phenotype can be investigated with ease, and NGS will allow regions of a gene not typically tested for mutations, such as deep intronic and promoter regions, to be analyzed on a routine basis. Here, we tested the analytical sensitivity and specificity of NGS for application in a clinical setting using previously identified simple and complex mutations.

The goal during a standard laboratory test development and validation process is to ensure the accuracy of the reported results. To achieve accuracy of results, laboratories have to ensure that every step of the testing process is carefully evaluated, and results documented to prove that a procedure works as expected and can consistently achieve the expected result. For a laboratory-developed test (LTD), laboratories are charged with establishing the following for the test: accuracy, precision, analytical sensitivity, analytical specificity, the reported range of test results, the test’s normal values, and the efficiency of the call rate for genotyping assays as indicated by the Center for Disease Control and Prevention, ACCE Model Process for Evaluating Genetic Tests as of January 3, 2010 (http://www.cdc.gov/genomics/gtesting/ACCE/). The analytical sensitivity of an assay is its ability to detect a low concentration of a given substance in a biological sample [10]. The sensitivity of NGS is vastly superior to Sanger sequencing and is capable of detecting mutant alleles as low >5%, as in mitochondria testing [11]. This extreme low level of the mutant allele will be undetectable by conventional Sanger sequencing and may not be confirmed as a “real” change. In our study, we are looking at two possibilities: equal proportion of both mutant and wild-type alleles, and either a mutant allele or a wild-type allele. The analytical specificity of an assay is its ability to identify only a specific substance [10]. In this study, we have assessed NGS for its application in clinical testing.

## Methods

### Validation samples selected for this study

For the first validation SOLiD sequencing run, we selected 20 samples that were referred to our laboratory for Sanger sequencing for a variety of different single-gene disorders. The selection of validation samples was based on the type of mutation present in the sample, the number of exons in the gene, and the complexity of the gene, which included % GC, sequence context around the mutation. The following genes were included: *ACADVL*, *BCKDHA*, *CBS*, *CFTR*, *DMD*, *GAA*, *GALC*, *GALT*, *GBA*, *GJB2*, *HEXB*, *IDUA*, *OPA1*, *RECQL4*, *SGSH*, *SMPD1,* and *ZEB2*. Samples selected for use in the validation of the SOLiD v3 instrument carried 119 changes consisting of 102 missense changes, seven deletions, nine duplications/insertions, and one indel mutation. These changes were initially identified by standard conventional Sanger clinical sequencing assays.

### DNA isolation and sample enrichment

Genomic DNA was purified from peripheral blood or saliva samples (DNA Genotek) using standard extraction conditions as recommended by the Puregene DNA extraction system (Qiagen). The coding region and at least 20 bp of the flanking intronic sequence were amplified using custom-designed primers (Additional file 1) using the FastStart Taq PCR system (Roche Applied Sciences). PCR products ranged in size from 250 bp to 750 bp. PCR amplifications were performed in 50-ul reactions using 50 ng of genomic DNA, 10X reaction buffer, 0.2 mM of each dNTP, 2 pM of each forward and reverse primer, and 2U of Taq polymerase. The cycling condition consisted of an initial denaturation at 95°C for 3 min, 10 cycles of step-down annealing, where there was a decrease of 0.5°C at each cycle following the initial condition of 1 min denaturation at 95°C, 1 min of annealing at 60°C, and extension for 1 min at 72°C. 25 cycles of minute denaturation at 95°C, 1 min of annealing at 55°C, and extension for 1 min at 72°C and a final 7-min extension at 72°C. After amplification the PCR products were visualized on a 2% agarose gel and purified with Millipore MultiScreen PCR UF 96-well plates (Millipore). Enriched amplicons were quantitated in triplicate using PicoGreen (Life Technologies) and pooled in equimolar amounts.

### Next-generation sequencing (NGS) analysis on an ABI SOLiD v3 sequencer

Each pooled sample was end-repaired (Epicenter Biotechnologies) and concatenated (New England BioLabs) using the manufacturer’s standard instructions. Results of concatenation were checked using an Agilent Bioanalyzer DNA 7500 chip (Agilent) to ensure that individual PCR fragments had been joined end to end to form a larger molecular weight product. Concatenated sample was then sheared randomly using Covaris S2 sonicator, and the sample was checked using an Agilent Bioanalyzer DNA 7500 chip to ensure that sheared sample was within 150 bp to 180 bp. Shearing concatenated sample ensures that we have even, non-biased coverage across the regions of interest . Sheared samples were then end-repaired and sequencing adaptor with unique barcode attached to each sample. An Agilent Bioanalyzer high-sensitivity chip was run to assess the success of adaptor ligation, as sample size should be increased by 90 bp after ligation, to a size range of 240 bp to 270 bp. Each sample was then amplified using Platinum Taq PCR system and SOLiD fragment library oligo kit (Life Technologies). Samples were then quantified using an Agilent Bioanalyzer high-sensitivity chip. Quantification of each sample was performed by calculating the area under the peak using the Agilent Bioanalyzer manual integration feature. Each sample is diluted to 1 ng/ul and all 20 individually barcoded samples are pooled together to create a single SOLiD library. Barcoding allows multiple small enriched targets to be combined and analyzed. The SOLiD library containing all 20 barcoded samples were diluted to 60 pg/ul, and emulsion PCR using the Solid ePCR kit (Life Technologies) was performed at two titration points (1pM and 1.5pM). Beads were purified and enriched for beads that had amplified template attached. Beads were then quantified using a NanoDrop and an estimated 15 million beads were used to perform a work flow analysis (WFA) on a quad on the SOLiD instrument. Approximately 15 million beads were deposited on a single quad on the glass slide (Life Technologies). Data generated on the WFA run were then used to determine the quality and quantity of beads present in the sample. Using quantification data from the WFA run, 60 million beads were then deposited onto a new quad, and a 50-bp barcoded fragment sequencing run was performed on the SOLiD v3 instrument.

### Data analysis

Data were analyzed using a software package that was commercially available: NextGENe™ (SoftGenetics LLC). Raw data from the 20 samples were analyzed in NextGENe™ according to the manufacturers’ standard analysis process. A single nucleotide polymorphism (SNP) detection and small and large indel-calling algorithm was run. Two projects were created per sample; one with the 50-bp reads from each individual sample was aligned back against reference sequence, which was downloaded from NCBI. The second was running up to four cycles of condensation for each sample to ensure that small and large indels were detected. Analysis on NextGENe™ was performed on a dual quad core running at 3.33 GHz desktop computer with 48 GB of RAM and 1 TB of storage.

### Mutation and polymorphism nomenclature

The reference sequence used for the 20 samples is as follows in Table 1. Nucleotide numbering reflects the cDNA numbering, with +1 corresponding to the A nucleotide of the ATG translation initiation codon in the reference sequence. The initiation codon is codon 1.

**Table 1:** Validation sample changes

**Gene**	**Reference**	**Change**	**Coverage**	**Phred-like confidence Score**	**% WT**	**% Mut**	**A%**	**C%**	**G%**	**T%**	**Ins%**	**Del%**
***ACADVL***	NM_000018.2	c.-63_-49dupGGGCGTGCAGGACGC										
		c.1375_1376insC	10663	31.5	NA	32					31.93	
		c.1504C > G (p.L502V)	9193	31	60	38	1.5	58.57	37.59	2.3	0.00	0.04
		c.1605 + 6 T > C	5733	19.7	68	28	1.71	28.14	2.62	67.54	0.00	0.00
***BCKDHA_1***	NM_000709.3	c.118dupC	21692	29.5	NA	19					19.21	
		c.370C > T (p.R124W)	18217	32.3	58	39	1.77	58.36	0.48	39.35	0.00	0.04
***BCKDHA_2***	NM_000709.3	c.972C > T (p.F324)	15574	30.7	6	91	1.44	5.91	1.36	91.29	0.00	0.01
		c.995 + 26C > T	18624	33.7	57	41	0.61	57.17	0.85	41.36	0.03	0.02
		c.995 + 49 G > A	23230	30.3	6	92	91.90	1.10	6.00	0.96	0.00	0.04
		c.996-33dupC	15037	30.8	NA	75					74.61	
***CBS***	NM_000071.2	c.959 T > C (p.V320A)	8715	24.4	7	91	0.48	90.82	1.63	7.05	0.00	0.02
		c.1080C > T (p.A360)	5125	27.3	53	46	0.76	52.55	0.62	46.07	0.00	0.00
***CFTR***	NM_000492.3	c.1408 G > A (p.M470V)	9356	30.4	60	38	38.44	0.46	60.04	0.99	0.00	0.07
		c.1521_1523delCTT	5843	22.3	NA	19	0.21	80.35	0.22	0.21	0	19.01
		c.2052_2053insA	7714	24	NA	26					25.68	
***DMD***	NM_004006.2	c.2645A > G (p.D882G)	2974	19.6	5	93	4.98	0.87	92.67	1.48	0	0
		c.5234 G > A (p.R1745H)	4289	23.4	4	93	93.38	1.19	4.13	1.31	0	0
		c.5326-22 G > T	162	9.6	3	91	3.7	1.85	3.09	91.36	0	0
		c.6290 + 27 T > A	4085	22.9	3	94	94.15	1.35	1.98	2.5	0	0.02
		c.8810 G > A (p.R2937Q)	3235	20.1	4	94	94.03	1.08	4.33	0.56	0	0
***GAA***	NM_000152.3	c.324 T > C (p.C108)	7078	24.7	5	87	4.46	86.61	3.45	5.47	0.00	0.01
		c.547-4C > G	15200	13.2	5	84	2.89	4.70	84.39	7.99	0.00	0.02
		c.596A > G (p.H199R)	11616	29.2	9	86	9.13	2.95	85.73	2.18	0.00	0.02
		c.668 G > A (p.R223H)	10344	31.4	5	93	92.62	1.14	4.89	1.31	0.00	0.04
		c.858 + 7_858 + 8insAGCGGGC	6175		NA	3					3	
		c.858 + 30 T > C	3431	24.8	5	90	1.40	90.38	2.97	5.25	0.00	0.00
		c.859-48 T > C										
		c.955 + 12 G > A	11315	23.4	4	90	89.60	3.64	3.84	2.90	0.00	0.02
		c.1203 G > A (p.Q401)	8487	26.1	8	79	79.42	5.35	8.27	6.96	0.00	0.00
		c.1327-18A > G	7377	26.7	9	82	8.70	5.29	81.81	4.19	0.00	0.01
		c.1438-19 G > C	2511	16.9	12	75	6.49	75.07	11.59	6.85	0.00	0.00
		c.1551 + 49C > A	18394	27.9	7	90	89.74	6.59	1.92	1.73	0.00	0.01
		c.1581 G > A (p.R527)	8283	26.9	70	28	28.05	1.05	70.14	0.76	0.01	0.00
		c.1802C > T (p.S601L)	6027	21.4	59	34	2.17	59.25	4.35	34.23	0.00	0.00
		c.1888 + 21 G > A	7667	30.3	56	42	41.99	0.93	56.15	0.94	0.00	0.00
		c.2040 + 20A > G	5633	27.8	7	91	6.57	1.67	90.96	0.80	0.00	0.00
		c.2133A > G (p.T711)	8402	29.4	60	39	60.05	0.39	39.20	0.35	0.00	0.01
		c.2331 + 20 G > A	11993	23	4	92	91.80	2.13	3.91	2.13	0.00	0.03
		c.2338 G > A (p.V780I)	8957	10.2	11	83	82.93	3.74	10.73	2.57	0.00	0.03
		c.2553 G > A (p.G851)	11663	21.7	5	94	93.78	0.90	4.54	0.77	0.00	0.00
***GALC_1***	NM_000153.2	c.328 + 19 T > A	107	0	66	34	33.64	0.00	0.00	66.36	0.00	0.00
		c.329-35 G > A										
		c.550C > T (p.R184C)	9215	19.6	39	54	2.59	38.77	4.75	53.88	0	0
		c.621 + 24 T > C	1178	17.1	82	16	1.61	15.87	0.34	82.17	0	0
		c.742 G > A (p.D248N)	15664	32.8	62	35	35.28	0.5	61.78	2.43	0	0.01
		c.1161 + 38 T > C	5842	29.6	56	42	0.77	42.23	0.74	56.23	0	0.03
		c.1586C > T (p.T529M)	15938	25.7	16	78	2.41	16.18	3.38	78.02	0	0.01
		c.1620A > G (p.T540)	25811	31.3	3	95	3.42	1.12	94.6	0.85	0	0.02
		c.1671-15C > T	17257	21.1	5	93	1.17	4.9	1.05	92.86	0	0.01
		c.1698A > T (p.V566)	41239	26.7	2	96	2.16	1.04	0.65	96.13	0	0.03
		c.1834 + 5C > G	10158	18.2	84	15	0.21	84.13	15.16	0.41	0.01	0.09
		c.1921A > G (p.T641A)	20556	23.6	2	97	2.13	0.88	96.5	0.49	0	0.01
***GALC_2***	NM_000153.2	c.328 + 19 T > A	193	0	85	15	15.03	0.00	0.00	84.97	0.00	0.00
		c.984 G > A (p.Q328)	17041	27.1	61	37	37	1.38	60.55	1.06	0	0
		c.1350C > T (p.S450)	17776	32.2	58	40	1.04	57.78	1.6	39.58	0	0.01
		c.1620A > G (p.T540)	25799	31.6	3	95	3.21	1.05	94.97	0.76	0	0.01
		c.1671-15C > T	22892	32.8	64	34	0.87	63.97	0.7	34.45	0	0.02
		c.1685 T > C (p.I562T)	30664	31.2	58	39	1.99	38.95	1.47	57.58	0	0.01
		c.1698A > T (p.V566)	42340	29.3	2	96	1.97	1.11	0.63	96.24	0	0.05
		c.1834 + 5C > G	10344	13.1	73	26	0.26	73.18	25.98	0.55	0	0.03
		c.1921A > G (p.T641A)	15648	23	2	96	2.19	1.04	96.42	0.35	0	0.01
***GALT***	NM_000155.2	c.776 G > A (p.R259Q)	27403	31.3	62	32	31.51	2.92	62.00	3.55	0.05	0.02
		c.817 G > C (p.D273H)	27824	32.3	61	38	0.68	37.71	60.85	0.73	0.00	0.03
***GBA_1***	NM_001005741.2	c.1225-34C > A	2621	22.8	2	96	96.26	2.21	1.14	0.38	0	0
		c.1226 A > G (p.N409S)	2864	20.4	62	36	61.91	0.8	36.03	1.26	0	0
		c.1448 T > C (p.L483P)	3331	26.4	60	38	0.99	38.04	0.9	60.07	0	0
		c.1483 G > C (p.A495P)	2569	16.3	53	44	1.01	44.45	53.41	1.05	0	0.08
		c.1497 G > C (p.V499)	2750	22.2	60	37	1.16	36.69	59.71	2.44	0	0
***GBA_2***	NM_001005741.2	IVS8-34C > A	2827	22	3	96	96.11	2.51	1.13	0.25	0	0
		c.1226 A > G (p.N409S)	3813	22.9	71	23	70.52	1.1	26.86	1.52	0	0
		c.1265-1317 del55	20	26.9	NA	25						25
***GJB2***	NM_004004.5	c.35dupG	33377	20.8	NA	18	0.74	0.33	1.28	96.64	17.63	1.01
		c.35delG	34879	29.7	NA	30						29.71
***HEXB***	NM_000521.3	c.185 T > C (p.L62S)	21320	33.2	8	86	3.63	86.44	2.33	7.59	0.00	0.02
		c.362A > G (p.K121R)	14201	27.9	66	32	65.63	1.11	31.86	1.39	0.00	0.00
		c.300-32C > T	792	20.5	80	17	1.77	80.05	1.26	16.92	0.00	0.00
		c.558 + 45 G > A	434	18.8	81	17	17.05	1.15	80.65	1.15	0.00	0.00
		c.1513C > T (p.R505W)	8306	30.9	62	37	0.52	61.77	0.92	36.78	0.00	0.01
		c.1619_1620ins22	27194		NA	67						
		c.1645 G > A (p.G549R)	33259	34.6	84	15	15.46	0.47	83.69	0.36	0.00	0.02
***IDUA***	NM_000203.3	c.99 T > G / p.H33Q	34	10.2	6	91	2.94	0.00	91.18	5.88	0.00	0.00
		c.208C > T (p.Q70X)	4106	9.4	49	46	2.65	49.05	1.90	46.40	0.00	0.00
		c.300-44C > T	10156	26.2	59	40	0.74	58.71	0.96	39.55	0.00	0.03
		c.314 G > A (p.R105Q)	10809	28.7	62	34	33.71	1.86	62.39	2.01	0.00	0.03
		c.543 T > C (p.N181)	12274	31.7	59	39	1.52	38.63	0.91	58.91	0.00	0.02
		c.590-45 G > C	11498	28.5	63	35	1.10	35.31	62.92	0.67	0.00	0.01
		c.590-8C > T	9516	30.8	63	34	1.46	63.36	1.04	34.03	0.00	0.12
		c.942 G > C (p.A314)	6026	20.9	63	34	2.09	29.67	66.69	1.51	0.00	0.03
		c.972 + 48A > G	1954	25.2	67	30	82.96	0.82	15.81	0.36	0.00	0.05
		c.973-45 G > C										
		c.1081 G > A (p.A361T)	9486	26.7	60	38	37.50	1.18	59.99	1.33	0.00	0.00
		c.1164 G > C (p.T388T)	3080	23.1	65	31	2.05	31.30	65.16	1.49	0.00	0.00
		c.1205 G > A (p.W402X)	2679	23.2	62	32	31.88	5.30	61.55	1.27	0.00	0.00
***OPA1***	NM_015560.2	c.93_96dupAAAA	179	0	NA	69					69.27	
		c.870 + 4 T > C	8330	28.8	3	97	2.23	89.09	1.81	6.87	0.00	0.00
		c.2808 G > A (p.A936)	8879	23	66	28	27.91	2.87	65.95	3.23	0.00	0.03
***RECQL4***	NM_004260.2	c.132A > G (p.E44)	5504	28.7	61	37	60.74	1.58	37.08	0.58	0	0.02
		c.274 T > C (p.S92P)	2505	11.9	14	75	4.79	74.61	6.43	14.17	0	0
		c.738C > T (p.S246S)	10356	27.9	76	24	0.3	75.69	0.32	23.68	0	0.02
		c.801 G > C (p.E267D)	5788	28.6	64	34	0.57	34.45	63.99	0.93	0	0.05
		c.1258 + 18 G > A	11609	27	65	31	30.65	2.64	64.82	1.86	0	0.03
		c.1621-15C > T	2331	26.6	63	34	1.12	63.32	1.54	34.02	0	0
		c.2297delC	8864	25	NA	94	0.37	3.77	1.55	0.17	0.01	94.14
		c.3014 G > A (p.R1005Q)	3027	27	50	48	47.9	1.06	50.21	0.83	0	0
		c.3127 T > C (p.L1043L)	13424	30.6	5	94	0.77	93.66	0.99	4.53	0	0.04
		c.3236 + 13C > T	1938	12	66	30	2.06	66.25	1.65	30.03	0	0
		c.3393 + 8C > T	2898	24.4	61	36	1.69	61.15	1.38	35.78	0	0
		c.3502 + 24 G > A	1106	20.2	64	30	29.57	2.35	64.2	3.8	0	0.09
***SGSH***	NM_000199.3	c.337_345delins11	7735	27.4	NA	23						22.82
		c.663 + 17 T > C	11973	29.6	59	39	0.93	38.84	1.24	58.96	0	0.04
		c.664-39_664-38delCT	153	30.6	NA	18	0	0	0	82.35	0	17.65
		c.664-36 T > C	149	30.5	82	18	0.00	18.12	0.00	81.88	0.00	0.00
		c.892 T > C (p.S298P)	12564	31.7	60	38	0.93	37.61	1.13	60.31	0	0.02
		c.1367 G > A (p.R456H)	8525	29.5	61	36	36.29	1.69	60.75	1.23	0	0.04
***SMPD1***	NM_000543.4	c.103CTGGCG[7]	5073	22.2	NA	88						88.07
		c.107 T > C (p.V36A)										
		c.785_807del23	10467	22.1	NA	20	0.06	0.16	0.01	79.68	0.01	20.09
***ZEB2***	NM_014795.3	c.2083C > T (p.R695X)	20800	24.9	75	24	0.51	75.22	0.4	23.88	0	0
		c.3067 + 6A > T	2632	24.3	60	39	59.95	0.68	0.53	38.79	0	0.04

Changes being assayed for in the 20 validation samples, along with corresponding coverage and reference sequence used during data analysis.

## Results

### Pooled PCR

Despite considerable work to ensure that each coding region of the entire library is represented equally during pooling, there is still great variability in the laboratory process that was hard to control. There seems to be lower coverage in the first coding exon of each of the 20 samples in this run, which may be due to the presence of higher GC content, whereas some additional exons gave a low coverage or no coverage (Table 2).

**Table 2:** GC content for first coding and (*) low-coverage exons (>20X coverage)

**Gene**	**Exon**	**GC content (%)**
***ACADVL***	1	70.2
***BCKDHA***	1	60.5
***CBS***	3	64.8
***CFTR***	1	56.3
***CFTR***	15*	39.1
***CFTR***	27*	52
***DMD***	1	32.2
***GAA***	2	66.8
***GALC***	1	73.0
***GALC***	17*	41.7
***GALT***	1	66.2
***GBA***	1	51.0
***GJB2***	2*	77.1
***HEXB***	1	71.5
***IDUA***	1*	76.7
***OPA1***	1	64.1
***OPA1***	17*	35.5
***OPA1***	23*	30.6
***RECQL4***	1	78.6
***SGSH***	1	75.7
***SMPD1***	1	69.0
***ZEB2***	2	49.4

### Target matched reads

In this run, a single quad generated 38,779,652 50-bp reads on the ABI SOLiD v3 instrument, which equated to 1,939 gigabases of data. Data generated from this run provided in excess of 1.9 million 50-bp reads per sample (Table 3). Approximately 53% of the 1.9 million 50-bp reads were good-quality data and mapped to the genes of interest, providing approximately an average of 71,000 reads per coding region and in excess of 9,800 reads per base. This indicates that our analytical specificity of good-quality reads is at 100% [12]. While we were able to identify all 119 expected changes as identified with our Sanger sequencing assay results, this data set had nine false-positive changes, which brought the analytical sensitivity of this study in at 92.7% [12].

**Table 3:** Run statistic

**Gene name**	**Total reads**	**Mappable reads**	**Aligned reads (%)**	**Reads/Exon**	**Ave. coverage/Base**	**Exon with min coverage**	**Min coverage**
***ACADVL***	2,459,557	1,352,756	55	39,971	10,278	2	957
***BCKDHA_1***	1,605,090	742,999	46.3	51,129	10,663	1	3695
***BCKDHA_2***	1,781,857	963,985	54.1	60,721	12,565	1	2390
***CBS***	2,032,870	1,120,111	55.1	35,875	8,729	3	1181
***CFTR***	2,025,300	1,042,746	51.5	23,522	4,895	15 & 27	0
***DMD***	1,862,128	996,238	53.5	8,385	1,821	2	68
***GAA***	1,956,406	1,013,418	51.8	26,236	5,431	18	1071
***GALC_1***	3,119,076	1,618,800	51.9	49,371	11,562	1	13
***GALC_2***	2,790,038	1,377,442	49.4	40,593	9,476	1	1428
***GALT***	1,795,122	1,086,049	60.5	60,858	15,566	8	5563
***GBA_1***	1,938,689	1,163,213	60	35,545	7,171	10	1277
***GBA_2***	1,793,280	1,075,968	60	32,945	6,641	10	541
***GJB2***	1,501,448	875,344	58.3	643,999	42,101	2	1
***HEXB***	1,851,105	897,786	48.5	34,570	7,641	12	1187
***IDUA***	1,275,727	637,864	50	22,550	4,978	1	4
***OPA1***	1,933,855	964,994	49.9	19,893	5,003	17 & 23	0
***RECQL4***	2,053,423	903,506	44	21,494	3,935	1	139
***SGSH***	1,845,441	992,663	53.8	47,870	9,990	1	3847
***SMPD1***	1,505,628	864,230	57.4	94,517	10,552	2	1986
***ZEB2***	1,653,612	1,036,815	62.7	82,365	8,394	8	1921
**Average**	1,938,983	1,036,346	53	71,620	9,870		

Run statistic from a single SOLiD v3 quad for all 20 barcoded samples.

### Data analysis

**Figure 1:**
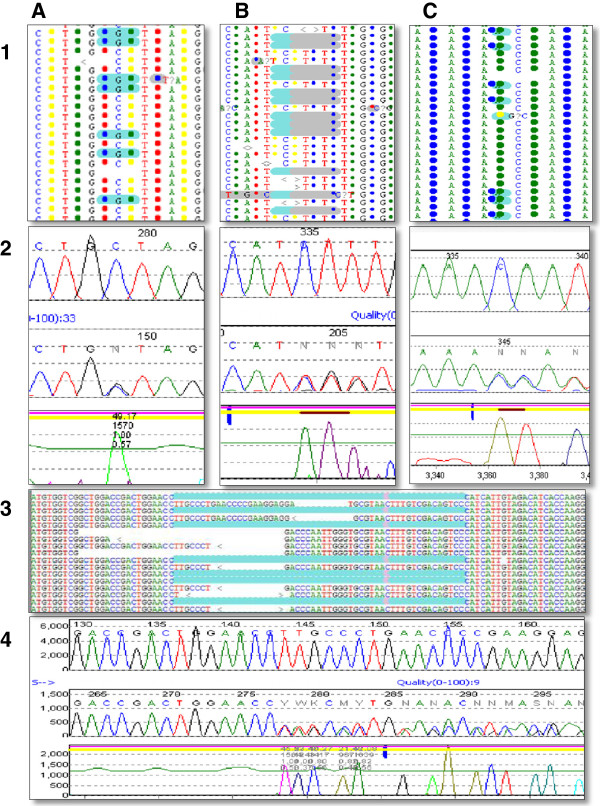
**Representative mutation as detected on Sanger and SOLiD platforms.** Panes 1**A** & 2**A** represent the SOLiD and Sanger data for *ACADVL* c.1504C > G (p.L502V) mutation. Panes 1**B** & 2**B** represent SOLiD and Sanger data for *CFTR* c.1521_1523delCTT mutation. Panes 1**C** & 2**C** represent SOLiD and Sanger data for *CFTR* c.2052_2053insA mutation. Panes 3 and 4 represent SOLiD and Sanger data for the *GBA* c.1265_1319del55 mutation.

Initial analysis with the NextGENe™ software was able to detect 119 out of the 119 expected changes (Table 4). Three changes (*IDUA* c.973-45 G > C, *OPA1* c.93_96dupAAAA and *SGSH* c.664-39_664-38delCT) missed during the initial analysis were complex changes or changes at the end of PCR fragments, where good-quality data were found to be discarded due to the initial software setting. The entire data set were subjected to analysis to determine the quality of each 50-bp read, with good-quality reads retained for additional analysis and bad-quality reads removed from analysis. The additional rounds of analysis performed on NextGENe™ used only good-quality reads for alignment for the three samples for which mutations were missed. This alternative strategy enabled the laboratory to detect the remaining three mutations that were missed in initial phases of the data analysis, and we were successful in detecting all 119 changes present in the data set. NextGENe™ was not only able to detect single nucleotide changes, such as *ACADVL* c.1504C > G (p.L502V), but also small deletions and insertion events, such as *CFTR* c.1521_1523delCTT and *CFTR* c.2052_2053insA. The real power of NextGENe software was its ability to detect larger deletions, duplications, and indels, such as *SMPD1* c.785_807del23, *SGSH* c.337_345delins11, and *GBA* c.1265_1317del55, using data generated from a 50-bp fragment sequencing run by applying a SoftGenetic’s propriety condensation algorithm, which enabled good-quality 50-bp fragment data to be lengthened and enabled the detection of larger size deletions and duplication events (Figure 1). This ability to detect the entire spectrum of mutations from single nucleotide changes to large deletions and duplications using the NextGENe™ software represents an important capability that a clinical laboratory has to have if they are to be able to offer clinical sequencing tests using next-generation sequencing data. This single run demonstrates that NGS software like NextGENe™ has matured sufficiently for use in a clinical environment and that next-generation sequencers, such as the ABI SOLiD, are ready to be deployed in clinical laboratories. While our data analysis pipeline was able to detect all 119 known changes, nine additional changes (six single nucleotide changes and three deletions) were also picked up. The laboratory was 100% concordant with the NGS data identifying all 119 known changes in the 20 samples. There were nine changes that were identified in the NGS data that were not identified in the Sanger sequencing data and that provided us with a 7.56% false-positive rate (Table 5).

**Table 4:** Number of changes

**Gene name**	**Sanger**	**NextGENe**	**% called**
***BCKDHA_1***	2	2	100%
***BCKDHA_2***	4	4	100%
***CBS***	2	2	100%
***CFTR***	3	3	100%
***DMD***	6	6	100%
***GAA***	20	20	100%
***GALC_1***	12	12	100%
***GALC_2***	9	9	100%
***GALT***	2	2	100%
***GBA_1***	5	5	100%
***GBA_2***	3	3	100%
***GJB2***	2	2	100%
***HEXB***	7	7	100%
***IDUA***	13	13	100%
***OPA1***	3	3	100%
***RECQL4***	11	11	100%
***SGSH***	6	6	100%
***SMPD1***	3	3	100%
***ZEB2***	2	2	100%
**Total changes**	119	119	100%

Summary of the number of changes picked up on the validation run.

**Table 5:** False-positive rate

**Change category**	**Sanger changes**	**Solid changes**	**No. of false positive**	**False positive rate**
**SNP**	102	108	6	5.88%
**Duplication / Insertion**	9	9	0	0.00%
**Deletion**	7	10	3	42.86%
**Indel**	1	1	0	0.00%
**Total (Overall)**	119	128	9	7.56%

Summary of false-positive rates per change category.

### Coverage

The coverage of each coding region ranged from 643,999 reads per exon for a small gene like *GJB2*, to the largest gene, which had an average of over 8,000 reads for the 79 coding regions in the *DMD* gene. For substitution changes, coverage ranged from 34 to 42340 reads. For deletions, the coverage ranged from 20 to 34879 reads. For duplications or insertions, the coverage ranged from 179 to 33377 reads. For the single indel mutation, coverage was 7735 reads (Table 1).

## Discussion

It is critical to ensure that samples selected for use in validation of NGS carried representative changes and mutations that a clinical laboratory expects to detect in real-world samples.

### NGS is able to detect complex mutations using targeted amplification

Genes selected included the *ACADVL, BCKDHA, CBS, CFTR, DMD, GAA, GALC, GALT, GBA, GJB2, HEXB, IDUA, OPA1, REQL4, SGSH, SMPD1* and *ZEB2* genes. Duchenne muscular dystrophy (DMD) is caused by mutations in the *DMD* gene, the largest human gene, spanning 2.2 Mb on the X chromosome [13,14]. Gaucher disease is an autosomal recessive disorder where mutations in the *GBA* gene result in a decrease in the activity of acid β-glucosidase. The *GBA* gene is an extremely difficult gene to perform diagnostic testing on, due to the presence of a pseudogene that is >98% identical to the active gene [15,16]. The *REQL4* gene has an atypical structure; it is a very compact gene of ~6.5 kb, where most of the introns are less than 100 bp in length. It is also highly repetitive and GC rich, making it difficult to amplify and sequence cleanly [17,18]. Other genes selected for inclusion in the validation run were mainly based on the changes they carry. One such example is a sample with two mutations in the *GJB2* gene. This sample carries a c.35delG on one allele and a c.35dupG on the second allele (Table 1). In conventional Sanger sequencing analysis, it is very difficult to interpret the data when there are two deletions at the same nucleotide position [19]. Both mutations in the *GJB2* gene were identified on the NGS run. NGS is able to sequence both strands independently, providing our laboratory with not only the genotype but also the data to determine which change is on which strand of the DNA.

### Target amplification method needs to be chosen carefully for NGS

In this study, we used a standard PCR approach to test the sensitivity and specificity of NGS. We faced many challenges during the initial startup phase in acquiring and deploying an NGS instrument in a clinical laboratory environment. Clinical laboratories routinely generate hundreds if not thousands of PCR reactions a day for use in Sanger sequencing, but this enrichment strategy would not work for NGS; it involves too many labor-intensive steps to accurately quantitate individual PCR amplicons before it can be pooled for use in the NGS chemistry pipeline. This labor-intensive manual process will raise costs and lengthen the time of the entire process. Laboratories will find it hard to continue to use standard Sanger sequencing enrichment techniques on a routine basis, because of the need to exploit the full capacity of the NGS instrument to minimize costs. On the SOLiD v3 instrument, we are able to interrogate up to 2.4 Mbp of a region of interest in a single quad. The cost in time and effort to generate individual PCR amplicons for an entire 2.4-Mbp region of interest is prohibitive and raises the chances that a mistake will occur. Even if long PCR techniques could be employed as the enrichment technique, it would require 240, 10-kb individual reactions to enrich for a 2.4-Mbp region.

It is clear that, to manage the workflow of a larger number of amplicons for gene panels, clinical laboratories will need to consider target enrichment methods, such as multiplex PCR (Fluidigm™), microdroplet-based PCR (RainDance™), or in solution-based PCR (Agilent SureSelect™). Jones et. al [20]. have recently demonstrated the use of microdroplet-based PCR for the testing of 25 genes for congenital disorders of glycosylation (CDG) in a clinical laboratory. In the work performed by Jones et. al., it was shown that even after using target enrichment methods, some exons fail to give adequate coverage and still need Sanger sequencing to complete the clinical test. Sanger sequencing will continue to play an important role in the clinical laboratory for assay completeness, both for sequencing low-coverage and difficult regions in a gene and for confirmatory studies once a mutation is identified in a proband and additional family members need to be tested. Given our initial approach of adapting the enrichment method used for standard Sanger sequencing, we have demonstrated any change within the boundaries of custom-designed primers flanking the region of interest (eg, exons) can be detected successfully.

### Coverage

Using coverage data as the sole indicator of whether a change was real is difficult. The nine false-positive changes that were picked up had a median coverage of approximately 400 reads and a mean of approximately 3,600 reads. As a contrast, confirmed changes had approximate median coverage of 5,300 reads and an approximate mean coverage of 7,000 reads. The numbers of reads for actual confirmed changes are approximately 15-fold higher compared to false-positive changes. As the number of reads for both confirmed and false-positive changes overlaps significantly, we are unable to use just the number of reads as the sole indicator. In this study, we see a great overlap in coverage between the number of reads for substitution mutations and with smaller insertion/deletion mutations. To detect larger deletions/duplications using NextGENe’s™ condensation function, the number of reads was effectively reduced. The GBA_2 sample, c.1265_1319del55 mutation had only 20 reads, compared to the GJB2 sample, which has a single base deletion, c.35delG mutation that had 34,879 reads. Similarly, the OPA1 sample, c.93_96dupAAAA mutation has only 179 reads compared to the GJB2 sample, c.35dupG mutation, which had 33,377 reads. In an effort to try to determine an appropriate coverage threshold, simulation experiments were run for mutation c.2052_2053insA in the *CFTR* gene. A varying number of reads that align to the region were randomly selected and used for analysis. We performed 80 simulations with the number of reads selected varying from 15 to 50 reads for every 10,000 reads. Coverage for the insertion varied from 8 to 43. For some of the simulations, NextGENe was able to detect the insertion with coverage as low as 8 reads. We chose 20 reads as the average threshold. Other groups have also expressed a similar viewpoint [21-25]. In work performed by De Leeneer K. et. al., the authors have performed a detailed analysis to determine the coverage needed during a NGS sequencing run given two variables (quality score of data and sequencing errors) to detect heterozygous changes. In their paper, they have determined that data with a quality score of 30 will require a minimum 18X coverage if sequencing error is at 15% [24]. Dohm J et. al. in their study found bona fide SNPs by applying high coverage of >20X [24].

### Confidence score

Software has a Phred-like confidence score calculated with a novel SoftGenetics algorithm. The software algorithm takes into account multiple variables to calculate a final probability that any one change is a true. A phred score of 10 means there is approximately a 1 in 10 chance that the change is the result of an error, while a phred score of 30 represents a 1 in 1000 chance that the change is an error. This Phred-like score gives us greater confidence in determining true and false-positive changes. In our study, we have seen real changes with Phred-like confidence scores averaging a score of 24 with a minimum score of 9.4 and a maximum score of 34.6 (Table 1). Some changes detected using the condensation algorithm does not have a Phred-like confidence score. Confidence score of nine and above along with coverage above 20X makes it more likely that a change is real.

### Proportion of bases

Another indicator is the relative proportion of mutant compared to the wild-type base. In one of the samples we ran, there is a heterozygous c.1504 C > G (p.L502V) missense mutation in the *ACADVL* gene. This mutation had 5869 reads showing an approximately equal proportion of the wild-type C allele (60%) compared to the mutation G allele (40%). Our validation data set suggests that real heterozygous calls should be present in the data in approximately equal proportion and can range as to as much as 70% wild-type to 30% mutant, whereas homozygous/hemizygous calls should consist almost exclusively of the mutant allele but can range as much as 20% wild-type to 80% mutant. The proportion of bases called will never be exact, due to the presence of nonspecific amplification that was sequenced and aligned back to the regions of interest. This is compounded by errors generated during next generation sequencing wet bench process and errors generated by the Solid instrument during sequencing.

### NGS pipeline in a clinical laboratory

Most clinical laboratories are very well equipped and accustomed to performing high-complexity testing that requires multiple steps. While most clinical laboratories will not find it difficult to perform the wet bench work required to perform a NGS run, it is a challenge to maintain the same level of consistency as could be achieved easily with a Sanger sequencing pipeline.

The current NGS pipelines involve many interdependent steps, and a major challenge faced by our laboratory was how to accurately and consistently quantitate small amounts of the enriched library that are present in each single step of the process. A subtle change in quantity could result in a bad library preparation and lead to a less than ideal data set, especially if loading the quad to its maximum capacity. Equal deep coverage of at least 20 reads per base across every region of interest is needed to ensure that all changes are picked up accurately by the laboratory.

### Changes in laboratory structure

Clinical laboratories often lack experienced bioinformatics staff and the necessary computing infrastructure within a clinical setup. There are only a few NGS 50-bp fragment analysis programs available on the market. The few that exist were developed for use by programmers and bioinformatics specialists. This dearth of software packages, which are both 'laboratorian' friendly and powerful enough to perform *de novo* detection of the entire mutation spectrum, hinders developments that would enable to use of NGS fragment capabilities to perform targeted resequencing projects. We selected SoftGenetics NextGENe™ software package as it is designed to detect the entire mutation spectrum, including small and large indels using data generated from a 50-bp fragment run. Our laboratory has demonstrated that we are able to leverage the power of SOLiD’s 50-bp fragment run to detect not only single nucleotide changes, but also small and large indels. This is possible due to a proprietary indel detection process called condensation, developed by SoftGenetics [26]. The condensation tool is used to polish and lengthen short sequence reads into fragments that are longer and more accurate. The short reads from the SOLiD System are often not unique within the genome being analyzed. By clustering similar reads containing a unique anchor sequence, data of adequate coverage are condensed; short reads are lengthened and instrument errors are filtered from the analysis. This stage helps to prepare data for analysis in applications such as SNP/Indel detection by statistically removing many of the errors, while maintaining true variations. The reads used for each condensed read are recorded to maintain allele frequency information. In addition, the condensation tool can be set to automatically run multiple cycles, further increasing the read lengths. Condensation operates without referring to a reference sequence. Reads are clustered using 12-bp anchor sequences within the reads. Each possible 12-bp sequence within the reads is considered for indexing. All reads containing this exact sequence are clustered together to form a group. The group of reads is further sorted by the flanking shoulder sequences, immediately upstream and downstream from the anchor sequence, into subgroups. A consensus read, generally 1.6 times the original read length, is created for each subgroup. By removing many low-frequency, biased calls and improving alignment accuracy by lengthening reads, the condensation tool is useful for preparing data prior to indel detection. NextGENe™ then aligns the consensus reads to the reference sequence. NextGENe™ can be run by a laboratory technician, which is an important consideration for a clinical laboratory. A laboratory technician who has been trained to analyze Sanger sequencing data does not necessarily have the programming skills to perform NGS analysis. Skilled professional programmers or bioinformatics specialists are needed to work in partnership with laboratory directors, genetics counselors, and clinicians to interpret the massive amount of data generated in a single NGS run.

Due to the immense capacity to generate data from a NGS platform, clinical laboratories will not perform single-gene analysis on the NGS platform. We are able to use the increased capabilities of the NGS platform by raising the number of genes being analyzed at a time. As the number of genes in a gene panel increases, the potential number of false positives identified will correspondingly go up. Clinical laboratories will deal with a larger number of false-positive changes in order to avoid missing any real disease-causing mutations. As with any clinical test, changes identified from a NGS platform will need to be confirmed using an alternative technology, such as Sanger sequencing. It is important that clinical laboratories perform such confirmation to determine the validity of calls generated by the NGS data. We have been able to identify three indicators (coverage of above 20 reads, confidence score of 30 and above and proportion of bases for heterozygotes that can range as skewed as 70% wild-type to 30% mutant and for homozygous as much as 20% wild-type to 80% mutant) to help to determine whether a change that is detected is real.

### Cost considerations when implementing NGS in a clinical laboratory

The cost of implementing a NGS system in a laboratory is not confined to the cost of the instrument package as provided by the manufacturer. There are many pieces of ancillary equipment required, and their availability will be critical to the success of the NGS setup in the laboratory. Equipment such as a powerful computer and secure data storage are required in the laboratory to handle the massive amounts of data. Cloud computing is an option that has emerged as NGS was developed over the last few years. While this is an alternative, the clinical laboratory will need to identify a secure HIPAA-compliant cloud provider that will be able to support clinical needs. While the cost of such a computer and storage cluster is reasonable, laboratories will need to budget additional funds to cover the purchase of such ancillary equipment.

## Conclusions

In conclusion, we have demonstrated that NGS technology is ready to be deployed in clinical laboratories. The analytical sensitivity achieved in our study was 92.7%, and was able to detect all 119 changes which were identified previously using Sanger sequencing. However, NGS and associated technologies are still in their infancy, and clinical laboratories will need to invest significantly in staff and infrastructure to build the necessary foundation for success. It has been suggested by many parties that the importance of targeted gene sequencing panels will decrease as the cost of NGS decreases. There is no need to just perform a targeted sequencing run when the same information can be extracted from a whole-exome or -genome analysis dataset. A recent study by Snyder et al. [27] suggests that, due to the size of the target that is being interrogated (exomes/genomes versus 2.4 Mbp), the lower depth of coverage reduces the sensitivity of variant detection. This affects the confidence of a clinical laboratory to detect all pertinent variants in our target genes. As such, targeted gene sequencing panels will continue to play an important role in clinical sequencing, until such time that whole exomes and genomes are able to reach the same level of high, even coverage as a targeted sequencing panel.

## Competing interests

The authors declare no competing interests.

## Authors’ contributions

EC participated in the drafting of the manuscript and participated in its design and coordination of work performed. CDS participated in data analysis and drafting of the manuscript. MRH conceived the study, participated in its design and helped to draft the manuscript. All authors read and approved the final manuscript.

## Supplementary Material

Additional file 1 Table of primers used in the amplification of the 20 validation samples. Click here for file
