# Supplementary material for: Assessment of clinical analytical sensitivity and specificity of next-generation sequencing for detection of simple and complex mutations
Source: BMC Genet. 2013 Feb 19;14:6. doi: 10.1186/1471-2156-14-6 (PMC3599218; doi:10.1186/1471-2156-14-6)
Supplement: Additional file 1 — Table of primers used in the amplification of the 20 validation samples. [file 1471-2156-14-6-S1.doc]

Supplemental Table: Primer Sequence of primers used in enrichment.

| **Primer Set** | **Size (bp)** | **Primer Name** | **Primer Sequence** |
| --- | --- | --- | --- |
| **ACADVL Ex1-2a** | 627 | ACADVL_Ex1_2Fa-2 | GTCCCATCCTATCCCATCAC |
|  |  | ACADVL_Ex1_2Ra-2 | GTACCTGGCACCAAGAGGGAG |
| **ACADVL Ex1-2b** | 385 | ACADVL_Ex1_2Fseq-2 | CAGCGGCGCCCGGAGAGATTC |
|  |  | ACADVL_Exon1-2R-2 | CGACTGAGCAGTAGGCAGGT |
| **ACADVL Ex3-5** | 592 | ACADVL_Ex3_5Fa-2 | CGAAACTAGGGGAAAGGTCAC |
|  |  | ACADVL_Ex3_5Ra-2 | CTCCAGAGCGTCATTCTTGG |
| **ACADVL Ex6** | 411 | ACADVL_Exon6F-2 | AAGAGCTGGTGGAGCCTGT |
|  |  | ACADVL_Exon6R-2 | GGGCAGGTGCTTAGAAGAGA |
| **ACADVL Ex7** | 536 | ACADVL_Exon7F-2 | CTTTGCACACCCCACTTCTT |
|  |  | ACADVL_Exon7R-2 | CAGTTCCCCCTTAACACCTG |
| **ACADVL Ex7a** | 273 | ACADVL_Ex7Fa-2 | CAGTGACAACCCCAGATTCC |
|  |  | ACADVL_Ex7Ra-2 | TAATCTGTGCCAAGCCCAGT |
| **ACADVL Ex8c** | 268 | ACADVL_Ex8Fc-2 | AAAGTAGCTCTCTCCCCAACA |
|  |  | ACADVL_Ex8Rc-2 | ATATTCAGGGCGTGGAGGAC |
| **ACADVL Ex8-9** | 617 | ACADVL_Exon8-9F-2 | CCAGGTGTTAAGGGGGAACT |
|  |  | ACADVL_Exon8-9R-2 | AAAGGCCACATTCAGTCCAC |
| **ACADVL Ex9** | 320 | ACADVL_Ex9Fa-2 | CCATTTCTCCCCTTCTCCTC |
|  |  | ACADVL_Exon8-9R-2 | AAAGGCCACATTCAGTCCAC |
| **ACADVL Ex10a** | 436 | ACADVL_Exon10F-2 | GTGCATAAGGAGCGAAGGAG |
|  |  | ACADVL_Exon10R-2 | GCTGGTAGGGGAGGAGTTTC |
| **ACADVL Ex10b** | 263 | ACADVL_Exon10F-2 | GTGCATAAGGAGCGAAGGAG |
|  |  | ACADVL_Exon10Rseq-2 | TTCCATTGTTGAGGATGTGC |
| **ACADVL Ex11** | 319 | ACADVL_Exon11F-2 | TATGCAAAACCCATCCCTCT |
|  |  | ACADVL_Exon11Ra-2 | GTGCTGCTGGGGGTTGTAG |
| **ACADVL Ex12-14** | 627 | ACADVL_Exon12-14F-2 | CTTGGAGATCTGGGTGATGAG |
|  |  | ACADVL_Exon12-14R-2 | CCTTTGTCCTATGGGGGAAG |
| **ACADVL Ex15-17** | 630 | ACADVL_Exon15-17F-2 | TGCAGGGCTGTATGGTAAGA |
|  |  | ACADVL_Exon15-17R-2 | CTGTTCATCTGTCCGGTAGG |
| **ACADVL Ex18-19** | 533 | ACADVL_Exon18-19F-2 | GGGGATTGTCAGTAAGTGAGC |
|  |  | ACADVL_Exon18-19R-2 | TGCTTTTGAAGTTGCGGTAG |
| **ACADVL Ex20** | 625 | ACADVL_Ex20Fa-2 | GAGAAAATGCTCTGTGACACCTG |
|  |  | ACADVL_Ex20Rb | TGAGGAAGCTGGGAGATTTG |
| **BCKDHA Ex1** | 154 | BCKDHA Ex1F-4 | gaccgctgagtggttgttag |
|  |  | BCKDHA Ex1R-4 | gcctggggcccaggtactc |
| **BCKDHA Ex2** | 316 | BCKDHA Ex2F-4 | tcgttctgatgcaggtggtctc |
|  |  | BCKDHA Ex2R-4 | gtggaggcacagatagacgt |
| **BCKDHA Ex3** | 181 | BCKDHA Ex3F-4 | ccacgtctatctgtgcctcc |
|  |  | BCKDHA Ex3Ra-4 | GCCCAAACACAGGTAGG |
| **BCKDHA Ex4** | 299 | BCKDHA Ex4Fa-4 | GGAGGACTGTGAATTCATGGAG |
|  |  | BCKDHA Ex4Ra-4 | CTGCTCCTGGAAGAACACT |
| **BCKDHA Ex5** | 338 | BCKDHA Ex5Fa-4 | GCCTGAGCTTTCCTGTCT |
|  |  | BCKDHA Ex5Ra-4 | AAGAGCTGAGGCCTGGA |
| **BCKDHA Ex6** | 373 | BCKDHA Ex6Fa-4 | CTGAGCCACGCTTGAGC |
|  |  | BCKDHA Ex6Ra-4 | CAGGAAGGTGGCAGGATT |
| **BCKDHA Ex7** | 308 | BCKDHA Ex7Fa-4 | GCCTCGTGCATGTTCCTTAT |
|  |  | BCKDHA Ex7Ra-4 | GCAACATCAGGTGATAAGGCC |
| **BCKDHA Ex8a** | 325 | BCKDHA Ex8Fc-4 | TCCCTAGTTCATCCCCCATC |
|  |  | BCKDHA Ex8Rc-4 | CCACAAATCCTTCCCAAGC |
| **BCKDHA Ex8b** | 237 | BCKDHA_Ex8Fseq-4 | ACCACAGCACCAGTGACGAC |
|  |  | BCKDHA_Ex8R-4 | TTCCACAAATCTTCCCAAG |
| **BCKDHA Ex9** | 351 | BCKDHA Ex9Fa-4 | GTGGTTAATTCCTTGCCAAGG |
|  |  | BCKDHA Ex9Ra-4 | AGTGGTGTGCTGTCAGG |
| **CBS Ex3** | 434 | CBS_Exon_3F | CTTCTCTGAGGGAGCAGAGC |
|  |  | CBS_Exon_3R | GGTGTCCAGGTAACAAACTCC |
| **CBS Ex4a** | 334 | CBS_Exon_4F | CCAGTTCTTCGAGTGTTGATTT |
|  |  | CBS_Exon_4R | TAAGGGATCCATCCCAGGAC |
| **CBS Ex4b** | 282 | CBS_Exon_4Fseq | CTCCTCTGTTTCAGGGCAAA |
|  |  | CBS_Exon_4R | TAAGGGATCCATCCCAGGAC |
| **CBS Ex5a** | 433 | CBS_Exon_5F | CCCTGGGGTGTATCAGGTAA |
|  |  | CBS_Exon_5R | GGAAGCTAGGTTGGGACACA |
| **CBS Ex5b** | 257 | CBS_Exon_5F | CCCTGGGGTGTATCAGGTAA |
|  |  | CBS_Exon_5Rseq | CGCTCAGCATCCTCAATCAT |
| **CBS Ex6-8** | 732 | CBS_Exon_6_8F | ATCCCACAGAACCCTCTTCC |
|  |  | CBS_Ex6_8Ra | AACGGAGGGAGGAAATTGTT |
| **CBS Ex9** | 283 | CBS_Exon_9F | TGGGCTGAGTGTGTTTTCAA |
|  |  | CBS_Exon_9R | GAATGACAGCTTTCAGCTCAG |
| **CBS Ex10** | 335 | CBS_Exon_10F | GCAGTTGTTAACGGCGGTAT |
|  |  | CBS_Exon_10R | CAGGCTCCCCAGTGTGAG |
| **CBS Ex11** | 285 | CBS_Exon_11F | AGGCTGTTCACCCTCTTGG |
|  |  | CBS_Exon_11R | GTCGGTGGCTGACTGAGG |
| **CBS Ex12** | 326 | CBS_Exon_12F | GTCTGACATGCTCCCATGC |
|  |  | CBS_Exon_12R | GTTCTCAGGTGAGGCGTGAG |
| **CBS Ex13a** | 257 | CBS_Exon_13F | GTGCGTGCCACTCAGCAG |
|  |  | CBS_Exon_13R | GGAGTGCTCTCCTGCCTGT |
| **CBS_Ex13b** | 464 | CBS_Ex13Fa | GTGTTTATCTGAGTGCCTGAGC |
|  |  | CBS_Ex13Ra | CTCTCCTGCCTGTCACACTG |
| **CBS Ex14** | 335 | CBS_Exon_14F | CCTCCTGCTGAGGTGCTG |
|  |  | CBS_Exon_14R | GCTGCCCTGTCCAGTGAC |
| **CBS Ex15a** | 336 | CBS_Exon_15F | GTAGGCTCGTGGCAGAGGAC |
|  |  | CBS_Ex15Rc | TTGAGCTGCCTGTAGGTGAC |
| **CBS_Ex15b** | 382 | CBS_Ex15Fa | GGTTTCTTTCTCCCCACCAG |
|  |  | CBS_Ex15Ra | CCTGTTTGAGCTGCCTGTAG |
| **CBS Ex16** | 313 | CBS_Exon_16F | CATCCCCTCTCACTCCACAG |
|  |  | CBS_Exon_16R | ACCACCCACTCCCCTAACAC |
| **CBS_Exon_17c** | 697 | CBS_Exon_17Fc | GGGGCTCATGTCAGAAGAAC |
|  |  | CBS_Exon_18R | CAATCACGCGTGTGTTTAGG |
| **CBS Ex17b** | 255 | CBS_Exon_18Fseq | AACCCACTGCCTCGTTCTC |
|  |  | CBS_Exon_18R | CAATCACGCGTGTGTTTAGG |
| **CF_Ex1** | 215 | CF Ex1F | GTAGTAGGTCTTTGGCATTAGGAGC |
|  |  | CF Ex1R | GCTTATTCCTTTACCCCAAACC |
| **CF_Ex2** | 275 | CF Ex2F | TGCCAGAAAAGTTGAATAGTATCAGA |
|  |  | CF Ex2R | AATATGTTTGCTTTCTCTTCTCTAAATAATTAA |
| **CF_Ex3** | 330 | CF Ex3F | GTGTGAATCAAACTATGTTAAGGGAAA |
|  |  | CF Ex3R | GGTTTCTTAGTGTTTGGAGTTGG |
| **CF_Ex4_1** | 481 | CF Ex4F | CTAAGAGTTTCACATATGGTATGACCC |
|  |  | CF Ex4R | GCATATTAAATTAATTTCAGCATTTATCC |
| **CF_Ex4_2** | 388 | CF Ex4Fseq | GATGAAAAGTCTTGTGTTGAAATTCTC |
|  |  | CF Ex4Rseq | TTGTACCAGCTCACTACCTAATTTATGA |
| **CF_Ex5** | 289 | CF Ex5F | ACTTAATAATGAATGCATAATAACTGAATTAGTC |
|  |  | CF Ex5R | AATTACTATTATCTGACCCAGGAAAACTC |
| **CF_Ex6a** | 324 | CF Ex6F | TGCTTTCTTTCATATATGATTGTTAGTTTC |
|  |  | CF Ex6R | GCAGTCCTGGTTTTACTAAAGTGG |
| **CF_Ex6b_1** | 363 | CF Ex7F | AAATATGACTTAAAACCTTGAGCAGTTC |
|  |  | CF Ex7R | TGCATGAATATTGACAGAACTTAAATG |
| **CF_Ex6b_2** | 327 | CF Ex7F | AAATATGACTTAAAACCTTGAGCAGTTC |
|  |  | CF Ex7Rseq | GATTGTCACAAACATCAAATATGAGG |
| **CF_Ex7_1** | 535 | CF Ex8F | GGCAGAAAGACTCTAGAGACCATG |
|  |  | CF Ex8R | CCAGAGAAATGCTAGGAAAAGTTTAA |
| **CF_Ex7_2** | 490 | CF Ex8F | GGCAGAAAGACTCTAGAGACCATG |
|  |  | CF Ex8Rseq | AGCTGGCAACTTTTATAACTTCCTAGT |
| **CF_Ex8** | 289 | CF Ex9F | GATGTAGCACAATGAGAGTATAAAGTAGATG |
|  |  | CF Ex9R | GTTATATCATCATTCTAATTTTATTCGCC |
| **CF_Ex9_1** | 511 | CF Ex10F | CAAGCATCTATTGAAAATATCTGACAA |
|  |  | CF Ex10R | CAGTGTTGAATGTGGTGCAAA |
| **CF_Ex9_2** | 375 | CF Ex10F | CAAGCATCTATTGAAAATATCTGACAA |
|  |  | CF Ex10Rseq | TAATGCTCATGTAAGAATTTCTCCAA |
| **CF_Ex10_1** | 378 | CF Ex11F | CACTTCTGCTTAGGATGATAATTGG |
|  |  | CF Ex11R | TATATGTAGACTAACCGATTGAATATGGAG |
| **CF_Ex10_2** | 285 | CF Ex11Fseq | TGATAATGACCTAATAATGATGGGTTT |
|  |  | CF Ex11Rseq | TTGGGTAGTGTGAAGGGTTCATAT |
| **CF_Ex11** | 304 | CF Ex12F | TGGTTAAAGCAATAGTGTGATATATGATTAC |
|  |  | CF Ex12R | CCAGAAACAGAATATAAAGCAATAGAGAA |
| **CF_Ex12_1** | 329 | CF Ex13-1F | TGTGGTGACCATATTGTAATGCA |
|  |  | CF Ex13-1R | CCATGCTACATTCTGCCATACC |
| **CF_Ex12_2** | 259 | CF Ex13-2F | TGTAGTGAACTGTTTAAGGCAAATCA |
|  |  | CF Ex13-2R | AGGTAAAATGCAATCTATGATGGG |
| **CF_Ex13_1** | 497 | CF Ex14-1F | GATAGAGATTATATGCAATAAAACATTAACAAA |
|  |  | CF Ex14-1R | GTAAGGGAGTCTTTTGCACAATG |
| **CF_Ex13_2** | 462 | CF Ex14-1Fseq | GCTAAAATACGAGACATATTGCAATAAA |
|  |  | CF Ex14-1R | GTAAGGGAGTCTTTTGCACAATG |
| **CF_Ex13_3** | 485 | CF Ex14-2F | TGGGGAAAAAAGGAAGAATTCT |
|  |  | CF Ex14-2R | TTACATGCTACATATTGCATTCTACTCA |
| **CF_Ex13_4** | 455 | CF Ex14-2F | TGGGGAAAAAAGGAAGAATTCT |
|  |  | CF Ex14-2Rseq | TGCATTCTGTGGGGTGAAA |
| **CF_Ex14a** | 274 | CF Ex15F | AATAAAACCACAATGGTGGCAT |
|  |  | CF Ex15R | GCATATATATGTATACATCCCCAAACTATCT |
| **CF_Ex14b** | 273 | CF Ex16F | GCAAAGGAAGATGAAATTGTGTG |
|  |  | CF Ex16R | GAAACAAAGTGGATTACAATACATACAAAC |
| **CF_Ex15** | 417 | CF Ex17F | TTAGACTCAAGTTTAGTTCCATTTACATGT |
|  |  | CF Ex17R | CCATTAGAAAACCAACAAAACCAC |
| **CF_Ex16_1** | 376 | CF Ex18F | TTTCTAAGTCTATCTGATTCTATTTGCTAATTC |
|  |  | CF Ex18R | CTATTTTGAAGGCTTTGGATAATTACA |
| **CF_Ex16_2** | 333 | CF Ex18F | TTTCTAAGTCTATCTGATTCTATTTGCTAATTC |
|  |  | CF Ex18Rseq | CAGGACTTCAACCCTCAATCAA |
| **CF_Ex17a** | 308 | CF Ex19F | GAAATAAATCACTGACACACTTTGTCC |
|  |  | CF Ex19R | GTAGATTAACAATAAAGAATCTCAAATAGCTCT |
| **CF_Ex17b** | 405 | CF Ex20F | CTAATTTAGTCTTTTTCAGGTACAAGATATTATG |
|  |  | CF Ex20R | AAAATTGATAACCTATAGAATGCAGCA |
| **CF_Ex18** | 295 | CF Ex21F | AATGTGATATGTGCCCTAGGAGAA |
|  |  | CF Ex21R | AGCTCAATATACGGTATATAGTTCTTCCTC |
| **CF_Ex19** | 454 | CF Ex22F | GCCCGACAAATAACCAAGTG |
|  |  | CF Ex22R | GCTAACACATTGCTTCAGGCTACT |
| **CF_Ex20** | 331 | CF Ex23F | CAATGGTTTTTATTGAAGTACAATACTGA |
|  |  | CF Ex23R | TATGAATTTCTTGAGTACAAGTATCAAATAGC |
| **CF_Ex21** | 478 | CFTR_Ex24Fa | CACAAGGGACTCCAAATATTGC |
|  |  | CFTR_Ex24Ra | GTCCAGTCAAAAGTACCTGTTGC |
| **CF_Ex22** | 331 | CF Ex25F | CCTGTGTTTATTTTTAGAATGTCAACTG |
|  |  | CF Ex25R | AGAAATATGTGTCACCATGAAGCA |
| **CF_Ex23** | 268 | CF Ex26F | CAATAGACATATTATCAAGGTAAATACAGATCA |
|  |  | CF Ex26R | CAATTTGCAGGAACTATCACATGT |
| **CF_Ex24** | 392 | CF Ex27F | GAGCCTGTGCCAGTTTCTGT |
|  |  | CFTR_Ex27Ra | CTTGTTTTCTGAGGCAGAGGTAAC |
| **DMD Ex1** |  | DMD Ex1F-2 | TAAAGTTTGAAGAACTTTTACCAGGTTTT |
|  |  | DMD Ex1R-2 | GTCACAAACTAAACGTTATGCCAC |
| **DMD Ex2** | 186 | DMD Ex2Fb-2 | GTAAAATATGAATTATATTTAAAGTTGCTTCCTAAC |
|  |  | DMD Ex2R-2 | ATAGTCCATTTTGAAAATTTCACAACTTAG |
| **DMD Ex3a** | 274 | DMD Ex3Fc-2 | TAATTATGCACACTAATTATCCTTAAATATAGCT |
|  |  | DMD Ex3R-2 | GTCAGTTTCTGGTCTGAAATTCTACTAAGT |
| **DMD Ex3b** |  | DMD Ex3_2F-2 | taatttcagtttgggaagcagc |
|  |  | DMD Ex3R-2 | GTCAGTTTCTGGTCTGAAATTCTACTAAGT |
| **DMD Ex4** | 215 | DMD Ex4F-2 | GGTTTCATTTCTAGTAGATTGTCGGTCT |
|  |  | DMD Ex4R-2 | CCAAAGCCCTCACTCAAACAT |
| **DMD Ex5** | 233 | DMD Ex5F-2 | TATTATTGCAACTAGGCATTTGGTCT |
|  |  | DMD Ex5R-2 | TTGTTTCACACGTCAAGGGTAA |
| **DMD Ex6** | 322 | DMD Ex6F-2 | CACTGAAGATCAAGGACATTCATATTTA |
|  |  | DMD Ex6R-2 | GTCATCAGAGTCTAAATCACCACTTTTACA |
| **DMD Ex7** | 287 | DMD Ex7F-2 | GCATGGAAGTAAATCTCATGGAAC |
|  |  | DMD Ex7R-2 | TTGTATTTTGTGTAGAAATGACAAGTCTCA |
| **DMD Ex8** | 340 | DMD Ex8F-2 | CCTTTAACTTTGATTTGTTCATTATCCTT |
|  |  | DMD Ex8R-2 | GTGCACGTAATACCTAAAAATGCATATA |
| **DMD Ex9** | 288 | DMD Ex9F-2 | GTAGTCCTTTCGGGTTACTTATGGTT |
|  |  | DMD Ex9R-2 | ACTGAAAAATTCAAGCAAGTAAAAGCA |
| **DMD Ex10** | 374 | DMD Ex10F-2 | CCAGTGGACAGTCCTAGCATTTTAA |
|  |  | DMD Ex10R-2 | AATTGAGGAAAAAGGATGACTTGC |
| **DMD Ex11** | 357 | DMD Ex11F-2 | CAAAACCACACCGATTTACCTAGA |
|  |  | DMD Ex11R-2 | ATCAAAATAATCACAAGCTTCCAAAA |
| **DMD Ex12** | 308 | DMD Ex12F-2 | TACATTGTGATGTTCAGTAATAAGTTGCTT |
|  |  | DMD Ex12R-2 | CATCAACCATGTCATCTGTGTTACTG |
| **DMD Ex13** | 282 | DMD Ex13F-2 | GAGATGTAGCAGAAATAAATTTCACCAT |
|  |  | DMD Ex13R-2 | TACTTTTCAAGTTATAGTTCTTTTAAAGGACATAT |
| **DMD Ex14_15** | 438 | DMD Ex14/15F-2 | TTTAATAAAACGTAGTTACCAATTGTTTGCTGATGC |
|  |  | DMD Ex14/15R-2 | AATAGTGATAATATACAGTACTGGGTTTTTATAAGA |
| **DMD Ex16** | 299 | DMD Ex16F-2 | CTATAGTGGTGTATGGAATGCAACC |
|  |  | DMD Ex16R-2 | TTAATGCAGGTTTAAAAAATCTCTGAGA |
| **DMD Ex17** | 294 | DMD Ex17F-2 | AGCAGTCTTTACTGAAGTCTTTCTAGCA |
|  |  | DMD Ex17R-2 | CTGCTGTAAATGAGTTTTCTCCACTT |
| **DMD Ex18** | 240 | DMD Ex18F-2 | AATATAGAAGAAAGAGATAATCAAGAAATAATGACT |
|  |  | DMD Ex18R-2 | GCACGGAGTTTACAAGCAGCA |
| **DMD Ex19** | 246 | DMD Ex19F-2 | TATAATTATTGTGTAGATTCACAGTCCTTGTATT |
|  |  | DMD Ex19R-2 | ATGAACCTATGTGTTTATCAAATCCCT |
| **DMD Ex20** | 364 | DMD Ex20F-2 | CAGATCATTTCTTTCAGTCTGTGG |
|  |  | DMD Ex20R-2 | TGGAATGCCAAGAAATACCTATTG |
| **DMD Ex21** | 294 | DMD Ex21F-2 | GCAAAATGTAATGTATGCAAAGTAAAC |
|  |  | DMD Ex21R-2 | ACAAATAACCATTTTGGAAAATGTCA |
| **DMD Ex22** | 251 | DMD Ex22F-2 | CATGGCAAAGTGTGAAACAATTAA |
|  |  | DMD Ex22R-2 | TATCAATGTGAATGCTTGATAAGC |
| **DMD Ex23** | 337 | DMD Ex23F-2 | CACTAAAACTCATCAATTATTATTCATCAATT |
|  |  | DMD Ex23R-2 | TATCGTTAGGGAAAAAACAAGTAAATAAAA |
| **DMD Ex24** | 233 | DMD Ex24F-2 | TGAATTGTGTTAAAAGTAATCAGCACAC |
|  |  | DMD Ex24R-2 | CTAACCAAATAATATTCATACAAAATTATTCATATT |
| **DMD Ex25** | 270 | DMD Ex25F-2 | CTAATATGTGGCAGTAATTTTTTTCAG |
|  |  | DMD Ex25R-2 | CATTAGGAAATCTTAGTTAAGTACGTTGAG |
| **DMD Ex26a** | 316 | DMD Ex26F-2 | TATAATAATAATGTTTCATCACTGTCAATAATCG |
|  |  | DMD Ex26R-2 | GTATACAACTTCAAGCATTGTTGCAT |
| **DMD Ex26b** |  | DMD Ex26F-2 | TATAATAATAATGTTTCATCACTGTCAATAATCG |
|  |  | DMD Ex26RSeq-2 | TTTACCTTCATCTCTTCAA |
| **DMD Ex27** | 297 | DMD Ex27F-2 | ATGCATTTTGGATGTAAAGTTATTTTCA |
|  |  | DMD Ex27R-2 | CACATATGACCATGTATTGACATATCATT |
| **DMD Ex28** | 256 | DMD Ex28F-2 | ATTTCACATTTACTTTTCTACCATAATATTTAATC |
|  |  | DMD Ex28R-2 | GGTACTTGACCTCTTTTAATACTGCATATAA |
| **DMD Ex29** |  | DMD Ex29F-2 | GATAATCCAATGTATTTAGAAAAAAAAGGAG |
|  |  | DMD Ex29R-2 | GTATCTGCTATACATTAATGCAAATTAGATTAAA |
| **DMD Ex30** |  | DMD Ex30F-2 | TTACAGAAAAGCTATCAAGAGTAAACATTTAAC |
|  |  | DMD Ex30R-2 | CAACATTTTGTTGAAGTAATAAAAACAAAAG |
| **DMD Ex31** | 231 | DMD Ex31F-2 | CTTGGAAAGTTAGTTGTTCTTTGTAGAG |
|  |  | DMD Ex31R-2 | ATAGACTGGAGTATAATGCCCAACGAAAACACG |
| **DMD Ex32** | 295 | DMD Ex32F-2 | TAGGACCAGTTATTGTTTGAAAGGC |
|  |  | DMD Ex32R-2 | TTACTTCTTAATGAGGAAAGTCAAGGG |
| **DMD Ex33** | 274 | DMD Ex33F-2 | ATTATGAAATAATTTAACTCTACTGATTATCATGTT |
|  |  | DMD Ex33R-2 | GTTGCTTTACAATTTATAAGGAAAGTGGA |
| **DMD Ex34** | 291 | DMD Ex34F-2 | TTATAACGAAATTTGAATTAAAGAGTAAACTAAA |
|  |  | DMD Ex34R-2 | AAAATCATATTATGTGTTTTCACGTATGTTC |
| **DMD Ex35** | 300 | DMD Ex35F-2 | AAGCATTAAATCTTAAGACTACA |
|  |  | DMD Ex35R-2 | CTTATGTATCTTTTTCTCGTGACAGAGAA |
| **DMD Ex36** | 245 | DMD Ex36F-2 | TTCTTTAAGAATATTGTCTAACCAATAATGC |
|  |  | DMD Ex36R-2 | AAGATGATTGAAGTAACTGGTGTACAATTTG |
| **DMD Ex37** | 286 | DMD Ex37F-2 | TCTATCTTGACCTTCATTAATTACTAACTTCA |
|  |  | DMD Ex37R-2 | CCTTCGCAAGAGACCATTTAGC |
| **DMD Ex38** | 243 | DMD Ex38F-2 | TGGTTTATGTTTCTAATAAAAAGTAATTTTGATT |
|  |  | DMD Ex38R-2 | TCTTTCCAAATATTTATTTCCACTCCTA |
| **DMD Ex39** | 267 | DMD Ex39F-2 | TTAACAATGTACAGCTTTTTAAAAACCAA |
|  |  | DMD Ex39R-2 | CATTATATTTTACCCTATATATTAAAAAAAAACCAC |
| **DMD Ex40** | 274 | DMD Ex40F-2 | CAGCCAGAAGTGCACTATACATATATATTG |
|  |  | DMD Ex40R-2 | CTTCACAGGTTAATTAAACTGTATAATAAAATCTG |
| **DMD Ex41** | 303 | DMD Ex41F-2 | GCAAGTCGGTTGATGTGGTTAG |
|  |  | DMD Ex41R-2 | GCCTCTGTTAATAGAGTAGTAGTTGCAAA |
| **DMD Ex42a** | 409 | DMD Ex42F-2 | AATGGAGGAGGTTTCACTGTTAGG |
|  |  | DMD Ex42Ra-2 | tgaagccaaccacactatcaagta |
| **DMD Ex42b** |  | DMD Ex42FSeq-2 | TTGTTCTTTTGTATATCTATACCAGCAC |
|  |  | DMD Ex42RSeq-2 | TACCTTCAGAGACTCCTCTTGC |
| **DMD Ex43a** | 279 | DMD Ex43F-2 | TATAGACAGCTAATTCATTTTTTTACTGTTTTAAA |
|  |  | DMD Ex43R-2 | TTCCCTGAAAACAAATCATTTCTG |
| **DMD Ex43b** |  | DMD Ex43FSeq-2 | ATATTACAGAATATAAAAGATAGTCTACAACAA |
|  |  | DMD Ex43R-2 | TTCCCTGAAAACAAATCATTTCTG |
| **DMD Ex44** | 268 | DMD Ex44F-2 | TCTATAATCTGTTTTACATAATCCATCTATTTTTCT |
|  |  | DMD Ex44R-2 | TAAAGAGTCCAGATGTGCTGAAGATAA |
| **DMD Ex45** | 284 | DMD Ex45F-2 | CTCAAATAAAAAGACATGGGGCTT |
|  |  | DMD Ex45R-2 | GCTTAAAAAGTCTGCTAAAATGTTTTCA |
| **DMD Ex46** | 265 | DMD Ex46F-2 | ATAGTTTGAGAACTATGTTGGAAAAAAAAA |
|  |  | DMD Ex46R-2 | AATAGATTCATATACTTCTTTATGCAAGCAG |
| **DMD Ex47** | 269 | DMD Ex47F-2 | CAAGGTAGTTGGAATTGTGCTGTAA |
|  |  | DMD Ex47R-2 | TACATACAAATACTTGCAACATTTAACACA |
| **DMD Ex48a** | 320 | DMD Ex48F-2 | TAAACATTTTGGCTTATGCCTTG |
|  |  | DMD Ex48Rc-2 | AAATGAGAAAATTCAGTGATATTGCC |
| **DMD Ex48b** |  | DMD Ex48F-2 | TAAACATTTTGGCTTATGCCTTG |
|  |  | DMD Ex48RSeq-2 | CCTACCTTAACGTCAAATGG |
| **DMD Ex49** | 217 | DMD Ex49F-2 | TAATTTTATTGCTAACTGTGAAGTTAATCTG |
|  |  | DMD Ex49R-2 | CAACAGGGGAAGCATAACCCATTATGA |
| **DMD Ex50** | 228 | DMD Ex50F-2 | TCATGAATTATCTTCAAAGTGTTAATCG |
|  |  | DMD Ex50R-2 | CATAGTTGCACTTTTGAACAAATAGCTAG |
| **DMD Ex51** | 383 | DMD Ex51F-2 | AATTGGCTCTTTAGCTTGTGTTTCTA |
|  |  | DMD Ex51R-2 | GAGTAAAGTGATTGGTGGAAAATCT |
| **DMD Ex52** | 235 | DMD Ex52F-2 | ATGTAAAAGGAATACACAACGCTGA |
|  |  | DMD Ex52R-2 | TATTGAAACTTGTCATGCATCTTGC |
| **DMD Ex53** | 341 | DMD Ex53F-2 | ATTTGTAAATAGAATTCCTCCAGACTAGC |
|  |  | DMD Ex53R-2 | ATCTATGGTATAATTTTATCAAATGTAACCAGT |
| **DMD Ex54** | 273 | DMD Ex54F-2 | AACTAGAGATTTCATAAAAAAAACTGACATTC |
|  |  | DMD Ex54R-2 | CCAGTTTCACCACCCCATTATTA |
| **DMD Ex55** | 350 | DMD Ex55F-2 | TTCACTAGGTGCACCATTCTGATAT |
|  |  | DMD Ex55Rb-2 | ACTATTTTGTTTTGTCCCTGGCTT |
| **DMD Ex56** | 285 | DMD Ex56F-2 | CATCGCTTGTTTCTTTTGTTTG |
|  |  | DMD Ex56R-2 | ATTTGGCCATTTTAATTCATTTGTG |
| **DMD Ex57** | 271 | DMD Ex57Fa-2 | CAATGGAATTGTTAGAATCATCAATTACAC |
|  |  | DMD Ex57R-2 | GTCACTGGATTACTATGTGCTTAACATG |
| **DMD Ex58** | 221 | DMD Ex58F-2 | ATTAATTTTGAGAAGAATGCCACAAG |
|  |  | DMD Ex58R-2 | CGTCACCACTGATCCTTCTATCA |
| **DMD Ex59** | 336 | DMD Ex59F-2 | TTGACAATGTTTAAAAAAAAAGAATGTG |
|  |  | DMD Ex59R-2 | CAGATTAGAAGCTCTTTTGAGTCTCTCA |
| **DMD Ex60** | 325 | DMD Ex60F-2 | TCAGTTCTTCTTGTTTTAAATATTCTCATCT |
|  |  | DMD Ex60Rb-2 | tcctatcctcacaaatattaccatgaa |
| **DMD Ex61** | 207 | DMD Ex61F-2 | ACATTGTTTTAATTGTTCCTCATTATATAGAA |
|  |  | DMD Ex61R-2 | ACTGTTATTCTTATTAATCAAGATGCAATAAAG |
| **DMD Ex62** | 170 | DMD Ex62F-2 | CCTGTTTGCGATGAATTTGACC |
|  |  | DMD Ex62R-2 | ACAGGTTAGTCACAATAAATGCTCTTTTA |
| **DMD Ex63** | 175 | DMD Ex63F-2 | TCTTGACTACTCATTGTAAATGCTAAAGTC |
|  |  | DMD Ex63R-2 | CTACTTTATCCTAAAGGTCACCTGTCATT |
| **DMD Ex64** | 178 | DMD Ex64F-2 | TTGTCTGTTATTTCTGATGGAATAACAA |
|  |  | DMD Ex64R-2 | CTAAGCAAAGACATAGTATCAAGATCTTCA |
| **DMD Ex65** | 321 | DMD Ex65F-2 | GACACTGAAAGGAAGGTTTTACTCTTT |
|  |  | DMD Ex65R-2 | CATTCTGTACGCTAAGCCTCCTG |
| **DMD Ex66** | 206 | DMD Ex66F-2 | AAGTGTTTACCCTCTAGGAAAGGGT |
|  |  | DMD Ex66R-2 | AGAACTAGGGTAATTAGCCAACATTAATAAA |
| **DMD Ex67** | 352 | DMD Ex67F-2 | GAATTGAGTTGGATGTCAGGTTC |
|  |  | DMD Ex67R-2 | CATACCTACTGCCTACTGAAGAGCT |
| **DMD Ex68a** | 310 | DMD Ex68F-2 | ATCTTGCCTTCTTTCCTTTCATCC |
|  |  | DMD Ex68R-2 | TGGCACAGGAGATAAAAGATCAA |
| **DMD Ex68b** |  | DMD Ex68F-2 | ATCTTGCCTTCTTTCCTTTCATCC |
|  |  | DMD Ex68RSeq-2 | TTGGTTCCTAATACCTGAATCCAAT |
| **DMD Ex69** | 232 | DMD Ex69F-2 | GTGTTCTTTGGGAATTTGATTCG |
|  |  | DMD Ex69R-2 | ACAAAACTGAAATTTATCCCAGGTG |
| **DMD Ex70** | 257 | DMD Ex70F-2 | TGGTCATTAGTTTTGAAATCATCCTG |
|  |  | DMD Ex70R-2 | CTGAGAGGAGTTCAAATATACATCAAACA |
| **DMD Ex71** | 143 | DMD Ex71F-2 | CGGCTGAGTTTGCGTGTGTC |
|  |  | DMD Ex71R-2 | AAGCGAGCGAATGTGTTGGT |
| **DMD Ex72** | 186 | DMD Ex72F-2 | GTGGGTTTTTTCTCCATTAATGG |
|  |  | DMD Ex72R-2 | TAGCTTTCCTTGGTTAGTTATTTCA |
| **DMD Ex73** | 184 | DMD Ex73F-2 | GTCTTGAATAGATTCTAAGACGTCACATAAG |
|  |  | DMD Ex73R-2 | ATCCCTCAAAGCAATTTCATTGT |
| **DMD Ex74** | 284 | DMD Ex74F-2 | CTAACCCCCAAAGCAAAATAAGG |
|  |  | DMD Ex74R-2 | ACTTTTCTATGTGTGCAAGTGTATGC |
| **DMD Ex75a** | 402 | DMD Ex75F-2 | TACCATGGTATATAAAATTTGGTGATGATAT |
|  |  | DMD Ex75R-2 | AGTTTTGTTTAAGAGGGAAAAATG |
| **DMD Ex75b** |  | DMD Ex75FSeq-2 | ATGCCAATAGGAATCTGCAAG |
|  |  | DMD Ex75RSeq-2 | ATCTCTCTCCTCACTTGCTCCA |
| **DMD Ex76** | 243 | DMD Ex76F-2 | AATTTATACATTTGTATGTTTATTATGAAAAGTAATT |
|  |  | DMD Ex76R-2 | TGTAATACGACTCTACCTTTCTTCAGACAACA |
| **DMD Ex77** | 213 | DMD Ex77F-2 | CCTTTAATATCTGTTTTCTATAAATGTAATTTTC |
|  |  | DMD Ex77R-2 | TAGGGAAGCGAGTGGCCTGA |
| **DMD Ex78** | 156 | DMD Ex78F-2 | GATATTTATGCATGTTTTTTTTCCCTT |
|  |  | DMD Ex78R-2 | ACATAAAAAGCAGGATGAGACAGACAG |
| **DMD Ex79** |  | DMD Ex79F-2 | ATGCTATCTATCTGCACCTTTTGTAA |
|  |  | DMD Ex79R-2 | GACTCCATCGCTCTGCCCAA |
| **GAA Ex2** | 716 | GAA-Exon 2F-2 | CTTTGAGAGCCCCGTGAG |
|  |  | GAA_Ex2R-2a-2 | GTGAGGTGCGTGGGTGTC |
| **GAA Ex3** | 308 | GAA Ex3F-2a-2-2 | TCTCTGGAGAGTAAGGTGGCTG |
|  |  | GAA Ex3R-2a-2 | AACGCGTGTGGCCCTC |
| **GAA Ex4_5a** | 493 | GAA-Exon 4-5F-2 | GGGTGCTCTCTGGGTGCT |
|  |  | GAA-Exon 4-5R-2 | CCTCATGCGGACCTCCAGT |
| **GAA Ex4_5b** |  | GAA Ex4F-2seq | CTCCGCCCTCCCAGG |
|  |  | GAA-Exon 4-5R-2 | CCTCATGCGGACCTCCAGT |
| **GAA Ex4_5c** |  | GAA-Exon 4-5F-2 | GGGTGCTCTCTGGGTGCT |
|  |  | GAA Ex5R-2seq | AGGGCCCCTCATGCG |
| **GAA Ex6_8** | 700 | GAA-Exon 6-8F-2 | CTGTGATTGGCCCATCTGT |
|  |  | GAA-Exon 6-8R-2 | CAGGATGACGCTGCACAGAGA |
| **GAA Ex9** | 289 | GAA-Exon 9F-2 | CCAGCCTCATCCTCTCACTG |
|  |  | GAA_Ex9R-2a-2 | TGGAGGCCTCTGCTTTCTAA |
| **GAA_Ex10a** |  | GAA_Exon10F-2a-2 | GCTCAGTGGGGCTTCCAT |
|  |  | GAA_Exon10R-2a-2 | AAGAGAAGCGCTGCTGGTAG |
| **GAA_Ex11a** |  | GAA_Exon11F-2a-2 | CAAGGCCTCTGGGGACTAC |
|  |  | GAA_Exon11R-2a-2 | TAAGTCTCCCAGGCCAGACA |
| **GAA Ex12** | 320 | GAA-Exon 12F-2 | CTGAAGAGGCAGCGACCTG |
|  |  | GAA-Exon 12R-2 | CGCTTTTCCTCCTCCCTGAG |
| **GAA Ex13_14** | 672 | GAA-Exon 13-14F-2 | TCATCCCAGAAAGCTCCTTG |
|  |  | GAA_Ex13_14R-2a-2 | ATTCCCAGGGGAGAGTCTTG |
| **GAA Ex15** | 388 | GAA-Exon 15F-2 | ATGCTGGGTGGCTGAGAAGT |
|  |  | GAA-Exon 15R-2 | CAGGCCCAAATGTTGTCTCAC |
| **GAA Ex16** | 378 | GAA-Exon 16F-2 | CCTTGAGCTCCAGAGAGCAG |
|  |  | GAA_Ex16R-2a-2 | CTGCTCTGGTCTCCCGTCT |
| **GAA Ex17** | 375 | GAA-Exon 17F-2 | GGAGATGGAGAGCGTGGTT |
|  |  | GAA-Exon 17R-2 | CCTGGCTGGCTCCTTGATAAC |
| **GAA Ex18** | 400 | GAA-Exon 18F-2 | CAGGTGTTCCTGCAGATCCT |
|  |  | GAA_Ex18R-2a-2 | TCCCCTCACCCCTTCTCA |
| **GAA Ex19** | 354 | GAA-Exon 19F-2 | ATGCCATCATGAGTCCCTGT |
|  |  | GAA_Ex19R-2a-2 | CGATCCCTGTGCCACTCT |
| **GAA Ex20** | 471 | GAA-Exon 20.1F-2 | ATGGAGCCGCCTTCTGAG |
|  |  | GAA-Exon 20.1R-2 | CGTGCTGGGAACAGATGGAG |
| **GALC Ex1** | 432 | GALC Ex 2F | CTCCTGCCCGTATCTATCGT |
|  |  | GALC Ex 2R: | ACTGGCACCCTAGGGGAAT |
| **GALC Ex2_3a** | 565 | GALC Ex 3-4F: | TGGTGTGCGTGAACACTGTA |
|  |  | GALC Ex 3-4R: | TGAAATCACAGTCCATATGCTGA |
| **GALC Ex2_3b** |  | GALC Ex3_4F Int Seq | TTTAGGCAACCTCCCGACTT |
|  |  | GALC Ex3_4 R_2 | AAAATGTTGATGGGAGAAATCC |
| **GALC Ex4** | 292 | GALC Ex 5F: | TGGGGAGTGAGATGGTCCTA |
|  |  | GALC Ex 5R: | TTCCACCAACACGATTCAGA |
| **GALC Ex5** | 330 | GALC Ex 6F: | CCAGCACCGTTATACCTCCT |
|  |  | GALC Ex 6R: | AGCCTCATGGCATAAAATGG |
| **GALC Ex6** | 265 | GALC Ex 7F: | AAAGATGATGAGAGAAATGGTATCG |
|  |  | GALC Ex 7R: | GGTATTTCCAACACAAATTTCC |
| **GALC Ex7** | 323 | GALC Ex 8F: | TTATCTATCTTGGTCATAAATTCAACA |
|  |  | GALC Ex 8R: | GGGGAGAAGGCAAGAAAAAG |
| **GALC Ex8a** | 428 | GALC Ex 9F: | ACCTGGAAGCTACCCAACTG |
|  |  | GALC Ex 9R: | TCATTGAATCTAGGCTGGAAGA |
| **GALC Ex8b** |  | GALC Ex9F Int Seq | TATCCTGGAACCCATTCAGC |
|  |  | GALC Ex9R_2 | GAGAGCTGCATGTATTTTGCT |
| **GALC Ex9** | 431 | GALC Ex 10F: | TTGGGTGATCCTTTTATTGTCA |
|  |  | GALC Ex 10R: | AAACACTGGCAAATCTTGCTT |
| **GALC Ex10a** | 429 | GALC Ex 11F: | TGAATCAGACTCAAATTGATATACAGC |
|  |  | GALC Ex 11R: | TGCTATGTTTTACGACTCATGG |
| **GALC Ex10b** |  | GALC Ex11F Int Seq | CCATTTTTGTCTTATTTGGAACTTT |
|  |  | GALC Ex 11R: | TGCTATGTTTTACGACTCATGG |
| **GALC Ex11a** | 279 | GALC Ex 12F: | TCTTGGGCATTAACTGTTGAACT |
|  |  | GALC Ex 12R: | GAACTACTGGCCTGTGACAGAA |
| **GALC Ex11b** |  | GALC Ex12F Int Seq | TCATAAACATTCTAAGTGCATACGG |
|  |  | GALC Ex12R_2 | CAGGGCCTCTGTCAATTCAT |
| **GALC Ex12** | 271 | GALC Ex 13F: | TCTTGCTGGTACTGATTTTGGA |
|  |  | GALC Ex 13R: | TGACATTTCTGTGCCCTTTT |
| **GALC Ex13** | 426 | GALC Ex 14F: | AAGGGCCTTGATATTGGTGA |
|  |  | GALC Ex 14R: | CATCATGCACCCAGTTTGAC |
| **GALC Ex14** | 416 | GALC Ex 15F: | AAGCTTTCAGAAGTGTTTCAGACAT |
|  |  | GALC Ex 15R: | GGTTCTTGAAATAGGAGGACCA |
| **GALC Ex15a** | 398 | GALC Ex 16F: | GTTTGGAAGCATGTGCTGTG |
|  |  | GALC Ex 16R: | CCAAACACCTTGGGTTGAAT |
| **GALC Ex15b** |  | GALC Ex16F_2 | TGCTGTGAAATGACATATCTGTACTA |
|  |  | GALC Ex16R Int Seq | TCAGTTACCTAAATCACCTGTAACC |
| **GALC Ex16** |  | GALC Ex 17F | TCAAGAACCCCACTGAATTATATG |
|  |  | GALC Ex 17R | CACACTTTCCCCCTCCTATTT |
| **GALC Ex17** |  | GALC Ex 18F | GGAATTGTGTTTTGCTGTGG |
|  |  | GALC Ex 18R | TGAAACAAGAATTGGCTCTGAA |
| **GALTEx1** | 108 | GALTEx1F-4 | ggggtggtgtggacggagaaa |
|  |  | GALTEx1R-4 | ggggacgaaagcttcctaag |
| **GALTEx2** | 169 | GALTEx2F-4 | tggtgggtgagacccaggag |
|  |  | GALTEx2R-4 | cctgtgggtggaggacagttct |
| **GALTEx3_4** | 219 | GALTEx3F-4 | gcctgtccagtctttgaagccc |
|  |  | GALTEx4R-4 | agggcgaaccccaatgc |
| **GALTEx5_6** | 339 | GALTEx5F-4 | gttcgccctgcccgta |
|  |  | GALTEx6R-4 | ccacagtgctggctcagac |
| **GALTEx7** | 122 | GALTEx7F-4 | gaggaaatatgccaatgatgtggagg |
|  |  | GALTEx7R-4 | tgctaaggcctcctagcaagtc |
| **GALT_Ex8** | 304 | GALTEx8F-4 | gctgaccacactccggc |
|  |  | GALT_Ex8R-4 | GGTCCAGATGCTGACCCTAC |
| **GALT_Ex9** | 265 | GALT_Ex9F-4 | CTAGGCACTGGATGGAGGTT |
|  |  | GALT_Ex9Rb-4 | TCACTAGGCTGAGCCCCAGG |
| **GALTEx10** | 154 | GALTEx10F-4 | gggagtaggtgctaacctggat |
|  |  | GALTEx10R-4 | cagaagtatcaggtgcctgcac |
| **GALTEx11** | 205 | GALTEx11F-4 | ccatgccaccattcttggcag |
|  |  | GALTEx11R-4 | tagcttttaaccctagatctctgaaggttc |
| **GBA Ex1** | 439 | GBA-2 Ex1F-2-2 | CCGGAATTACTTGCAGGGC |
|  |  | GBA-2 Ex1R-2 | CCCGGTCTCCCACATTCA |
| **GBA Ex2** | 320 | GBA-2 Ex2F-2 | CAGCTAAGCCCTGCCCAG |
|  |  | GBA-2 Ex2R-2 | ACAAAATCCTCACCCCAAAGTT |
| **GBA Ex3_4** | 609 | GBA-2 Ex3_4F-2 | GTGAGGAATTTTGAAACCGTGTT |
|  |  | GBA-2 Ex3_4R-2 | ACGAAAAGTTTCAATGGCTCTATG |
| **GBA Ex5_6** | 684 | GBA-2 Ex5_6F-2 | TCCCTTTGGCCCTGACTC |
|  |  | GBA-2 Ex5_6R-2 | CAGATCAGCATGGCTAAATGG |
| **GBA Ex7** | 390 | GBA-2 Ex7F-2 | GAGCCACCACACCCAGC |
|  |  | GBA-2 Ex7R-2 | GAGAAATCGCTCTAAGTTTGGGA |
| **GBA Ex8** | 562 | GBA-2 Ex8F-2 | CGTGTAGTCCCAGCTACTCAGG |
|  |  | GBA-2 Ex8R-2 | CTTCTGTCAGTCTTTGGTGAAACTAGTA |
| **GBA Ex9** | 572 | GBA-2 Ex9F-2 | ATTCCCTATCTTCCCTTTCCTTC |
|  |  | GBA-2 Ex9R-2 | GGTGCAAAAGGGGATGG |
| **GBA Ex10_11** | 501 | GBA-2 Ex10_11F-2 | CGTGGGTGGGTGACTTCTTA |
|  |  | GBA-2 Ex10_11R-2 | TCACACTGGCCCTGCTGT |
| **Cx26 Ex2a** | 800 | Cx26 Ex2AF-4 | AGGAAGAGATTTAAGCATGCT |
|  |  | Cx26 Ex2AR-4 | GTCGTACATGACATAGAAGACGT |
| **Cx26 Ex2b** | 800 | Cx26 Ex2BF-4 | CTGCAGCTGATCTTCGTG |
|  |  | Cx26 Ex2BR-4 | TGGAGTTTCACCTGAGGC |
| **IDUA Ex1** | 522 | IDUA Ex1 F-6 | GTCATCGGTCCTCAGAGCAG |
|  |  | IDUA Ex1 R-6 | GCTCCGGTCTCTGAAGCTCT |
| **IDUA Ex2** | 392 | IDUA Ex2 F-6 | GGCTTGAACGTGTGTGTCAG |
|  |  | IDUA Ex2 R-6 | CCAGCAAGGACACGCTCT |
| **IDUA Ex3_4** | 632 | IDUA Ex3-6 F-6 | AGTCCTGTGTGGCACCTTG |
|  |  | IDUA Ex3-6R-6 | TGCCCACCAATGTATCTCCT |
| **IDUA Ex5_6** | 623 | IDUA Ex5-6 F-6 | AGTCAGACGCCCTTCATCAC |
|  |  | IDUA Ex5-6 R-6 | CAGCACCACCAGGGTCAG |
| **IDUA Ex7_8** | 864 | IDUA Ex7-8 F-6 | CCACGACGGTACCAACTTCT |
|  |  | IDUA Ex7-8 R-6 | CTCCCCTTGGTGAAGGAGTC |
| **IDUA Ex9** | 439 | IDUA Ex9Fb-6 | TGGGGACTCCTTCACCAAG |
|  |  | IDUA Ex9Rb-6 | TGACACTCAGGCCTCGGCTC |
| **IDUA Ex9seq** |  | IDUA Ex9Fseq-2 | GCCCCGCAGATGAGGA |
|  |  | IDUA Ex9-10 R-6 | GGTCCTCAGGGTTCTCCAG |
| **IDUA Ex9long** |  | IDUA Ex9Fb-7 | TGGGGACTCCTTCACCAAG |
|  |  | IDUA Ex9-10 R-6 | GGTCCTCAGGGTTCTCCAG |
| **IDUA Ex10** | 507 | IDUA Ex10Fb-6 | TGCTGATCTACGCGAGCGAC |
|  |  | IDUA Ex9-10 R-6 | GGTCCTCAGGGTTCTCCAG |
| **IDUA Ex11_12** | 511 | IDUA Ex11-12 F-6 | GTGTGGGTGGGAGGTGGA |
|  |  | IDUA Ex11-12 R-6 | GCAAGTGGCCCGAGTGAC |
| **IDUA Ex13** | 328 | IDUA Ex13 F-6 | CTTGAGGGAATGAGGCTGTG |
|  |  | IDUA Ex13 R-6 | CCTGACCCCAGGCTTCTC |
| **IDUA Ex14** | 324 | IDUA Ex14 F-6 | CGAGAAGCCTGGGGTCAG |
|  |  | IDUA Ex14 R-6 | GTGATGGGAGGGCAGCAT |
| **OPA1Ex1** | 314 | OPA1Ex1F-2 | acttcctgggtcattcctgg |
|  |  | OPA1Ex1R-2 | tctgggaattctccaactgc |
| **OPA1Ex2** | 520 | OPA1Ex2F-2 | tgctcttttaatgccatttcc |
|  |  | OPA1Ex2R-2 | catccaattgtattccactacacaa |
| **OPA1Ex3** | 478 | OPA1Ex3F-2 | tgcttgtttgctgagaccac |
|  |  | OPA1Ex3R-2 | tggcagctgtggtaaaattatg |
| **OPA1Ex4a** | 382 | OPA1Ex4-2F-2 | ttttgtagtggttgtcatgagg |
|  |  | OPA1Ex4-2R-2 | aaaaatgtcctgtttttcattgg |
| **OPA1Ex4b** | 284 | OPA1Ex4bF-2 | gccctatcgtaatatgaaatctga |
|  |  | OPA1Ex4bR-2 | cagcattataaatttggtctgcaa |
| **OPA1Ex5a** | 257 | OPA1Ex5F-2 | taggctgttgacatcactgg |
|  |  | OPA1Ex5R-2 | tatttgcttctcagatgttatca |
| **OPA1Ex5b** | 333 | OPA1Ex5bF-2 | accatccctccctagcttaca |
|  |  | OPA1Ex5bR-2 | tccatgaacagattgaggtgac |
| **OPA1Ex6** | 265 | OPA1Ex6F-2 | aaaaatttaacttgctgtacattctg |
|  |  | OPA1Ex6R-2 | caccttccaaattttgctctg |
| **OPA1Ex7** | 423 | OPA1Ex7F-2 | ttcctaatgttttcgtagatgcttt |
|  |  | OPA1Ex7R-2 | ctccatcctccaagcacatt |
| **OPA1Ex8** | 399 | OPA1Ex8-2F-2 | caatgattatggaaaaacaatttga |
|  |  | OPA1Ex8-2R-2 | ttgcttaagacattacttggaacat |
| **OPA1Ex9** | 288 | OPA1Ex9F-2 | agagcagcattacaaataggtttt |
|  |  | OPA1Ex9R-2 | caggtttccctgaagcagtt |
| **OPA1Ex10_11** | 512 | OPA1Ex10F-2 | gcaatgcagtagccctgtct |
|  |  | OPA1Ex11R-2 | agcagcaatctaaacatcaatacct |
| **OPA1Ex12_13** | 528 | OPA1Ex12F-2 | agcgtcttatctgaatggatga |
|  |  | OPA1Ex13R-2 | cgaagagaaggcaaaaatgc |
| **OPA1Ex14** | 330 | OPA1Ex14F-2 | tgctataatgtagacacaggg |
|  |  | OPA1Ex14R-2 | aacaaatccctatcacagctg |
| **OPA1Ex15_16** | 438 | OPA1Ex15F-2 | agcattattttgctttctaaattgt |
|  |  | OPA1Ex16R-2 | tgaaaacagttcaatttaagctactc |
| **OPA1Ex17** | 318 | OPA1Ex17F-2 | ctgttagcaagcacattcgc |
|  |  | OPA1Ex17R-2 | tatggatgccaaagattgcc |
| **OPA1Ex18** | 349 | OPA1Ex18F-2 | ttcagttaatacagaggatatg |
|  |  | OPA1Ex18R-2 | gataactgctcctagagatg |
| **OPA1Ex19** | 314 | OPA1Ex19F-2 | cagcctagtcaaaaacctccc |
|  |  | OPA1Ex19R-2 | caaggcaacaataaatcactgc |
| **OPA1Ex20** | 344 | OPA1Ex20F-2 | taatgatacttcagtcaagctg |
|  |  | OPA1Ex20R-2 | aaaattcacagctcctactcc |
| **OPA1Ex21** | 434 | OPA1Ex21F-2 | tggcttgagctcgtgttatt |
|  |  | OPA1Ex21R-2 | gggaaatattaaagggcagattac |
| **OPA1Ex22** | 289 | OPA1Ex22F-2 | tttttccatatttactaagctgtcaa |
|  |  | OPA1Ex22R-2 | gactccttcaccactgtgaactc |
| **OPA1Ex23** | 266 | OPA1Ex23F-2 | ttgaaatagttaagaaagcaagacca |
|  |  | OPA1Ex23R-2 | tgcctgaattaaaatgaacaaaa |
| **OPA1Ex24** | 501 | OPA1Ex24-2F-2 | ggggtgctgtgttctttctt |
|  |  | OPA1Ex24-2R-2 | tccttctcttcacgcaatca |
| **OPA1Ex25** | 381 | OPA1Ex25F-2 | ccttgcttttgcttttaattaacttt |
|  |  | OPA1Ex25R-2 | tccccagatgatcaaaggac |
| **OPA1Ex26** | 283 | OPA1Ex26F-2 | gtagttgtatgtgtttacgatg |
|  |  | OPA1Ex26R-2 | ggcatccttctattaaatatgg |
| **OPA1Ex27** | 430 | OPA1Ex27F-2 | ggtttcatgctgataaaactactgt |
|  |  | OPA1Ex27R-2 | gcccagttccttgtaacttttc |
| **OPA1Ex28** | 322 | OPA1Ex28-2F-2 | tcccgcaaatagttaagtatacca |
|  |  | OPA1Ex28-2R-2 | catgccattgaaattacagca |
| **RECQL4_Ex1** | 587 | RTS_1F | GCGGGAGATTCGCTGGAC |
|  |  | RTS_1R | TCTTCACTTTGCCCAGTCCC |
| **RECQL4_Ex2** | 512 | RTS_2F | TCCTCCCACTTCCCTGTTTG |
|  |  | RTS_2R | GAGAAGCTCCCTGAAGACTCGT |
| **RECQL4_Ex3** | 691 | RTS_3F | AGTGAGTCCACGCTAGGTCCAC |
|  |  | RTS_3R | GATGCTGACTTCTTGGAAGGCT |
| **RECQL4_Ex4** | 602 | RTS_4F | CAGAGGAATCACAACTTCTGATCC |
|  |  | RTS_4R | GGTCACTGGGCGGGAAATAC |
| **RECQL4_Ex5** | 437 | RTS_5F | ACGTACGGCTCAACATGAAGC |
|  |  | RTS_5R | GTACCTGGAAGGCCTGTTGC |
| **RECQL4_Ex6** | 606 | RTS_6Fnew | TCTTGTTTCCTGAACGAGCAGTT |
|  |  | RTS_6R | ACACAGATGTTGATCACCATGACTT |
| **RECQL4_Ex7** | 643 | RTS_7F | GGTTCCTTCACCACAACCTGTA |
|  |  | RTS_7R | GAGGCCACAGACACCTTGAAG |
| **RECQL4_Ex8** | 574 | RTS_8F | CAAGGGCAACTGCTGCTTG |
|  |  | RTS_8R | ACGTGTACCTGGGCTGCC |
| **RECQL4_Ex9** | 623 | RTS_9F | CATACACTCGGGCATGACCAG |
|  |  | RTS_9R | GGTCCCCAGAGCACACACA |
| **RECQL4_Ex10** | 682 | RTS_10Fnew | GCCTCACACCACTGCCGCCTCTGG |
|  |  | RTS_10Rnew | GACAGGCAGATGGTCAGTGGGATG |
| **RECQL4_Ex11a** | 617 | RTS_11F | CTCCTCATCAGGCACTGTTGAC |
|  |  | RTS_11R | GGGCAACTTTCATGAGGGTG |
| **RECQL4_Ex11b** | 537 | RTS_11F | CTCCTCATCAGGCACTGTTGAC |
|  |  | RTS_11Rseq | CTGCAGGAAGAGGTGGCA |
| **RECQL4_Ex12a** | 453 | RTS_12F | GCCACCTCTTCCTGCAGCC |
|  |  | RTS_12R | GTGGCTTACCCCAGGTTCC |
| **RECQL4_Ex12b** | 412 | RTS_12Fseq | CACACTGCCAGTGCTCGAG |
|  |  | RTS_12R | GTGGCTTACCCCAGGTTCC |
| **RECQL4_Ex13** | 533 | RTS_13F | CAAGAGGCTGAGCAGCTTAGC |
|  |  | RTS_13R | CACCAGCTTGACCATGTCAAA |
| **RECQL4_Ex14** | 659 | RTS_14F | CGAGACTTTGCTGTGCTACCTG |
|  |  | RTS_14R | TGTGAAAGGCCTGGAAGGTT |
| **RECQL4_Ex15** | 543 | RTS_15F | TGCTTGTGGAGTTCAGTGAGC |
|  |  | RTS_15R | GACAGGCACATCAGGCTTCC |
| **RECQL4_Ex16** | 568 | RTS_16F | TAAGGCCAGGCAGCTCATC |
|  |  | RTS_16R | TCAGTCACTGCCCTAGCCTCT |
| **SGSH Ex1** | 262 | SGSH Ex1Fb-5 | GTCTGGCGCGCACGTGAC |
|  |  | SGSH Ex1Rb-5 | AGGGCACACTAGGGTCAGCA |
| **SGSH Ex2** | 348 | SGSH Ex2F-5 | CCCTCACTCCCAGTGCTG |
|  |  | SGSH Ex2R-5 | GGATGGGAGACGTGGCA |
| **SGSH Ex3_4** | 719 | SGSH Ex3_4F-5 | TGGGGATGGGAGGGC |
|  |  | SGSH Ex3_4R-5 | CATCCCGCCGGAAGAC |
| **SGSH Ex5_6** | 736 | SGSH Ex5_6Fa-5 | GGTTGACTTCCAGATACAGAGGAG |
|  |  | SGSH Ex5_6Ra-5 | GTGACCTAAGAGGGCGCTG |
| **SGSH Ex7** | 466 | SGSH Ex7Fa-5 | GGAAAGCGCTGGAGCC |
|  |  | SGSH Ex7Ra-5 | ACCCAGATGATATGAGAGCCAC |
| **SGSH Ex8a** | 816 | SGSH Ex8Fb-5 | CGCTGCTGATTGGATTGGAGA |
|  |  | SGSH Ex8intR-5 | GTAGTAGTAATGACGGAGGTCCTTGTAC |
| **SGSH Ex8b** | 433 | SGSH Ex8intF-5 | TCCCATCGACCAGGACTTC |
|  |  | SGSH Ex8Rb-5 | GTGTGGACGGAAGGGCTGTTG |
| **SMPD1 Ex1a** | 558 | SMPD1_Ex 1.1F | CTGGTGACCTCAGGGAGAGT |
|  |  | SMPD1_Ex 1.1R | GATGTAACCTGGCAGGATGG |
| **SMPD1 Ex1b** |  | SMPD1_Ex 1.1Fseq | AGGGGCGGAGCTGCT |
|  |  | SMPD1_Ex 1.1R | GATGTAACCTGGCAGGATGG |
| **SMPD1 Ex1c** | 357 | SMPD1_Ex 1.2F | CCCGGACTCCTTTGGATG |
|  |  | SMPD1_Ex 1.2R | TTGTAGATGCCACCCTCTCC |
| **SMPD1 Ex1d** |  | SMPD1_Ex 1.2F | CCCGGACTCCTTTGGATG |
|  |  | SMPD1_Ex 1.2Rseq | AGCCCAGCGCACCAG |
| **SMPD1 Ex2a** | 580 | SMPD1_Ex 2.1F | CCAGCCCCAGTTTGGAAAT |
|  |  | SMPD1_Ex 2.1R | CCACTCAACAGGCTCTCCAG |
| **SMPD1 Ex2b** | 555 | SMPD1_Ex 2.2F | TGACTGTGCAGACCCACTGT |
|  |  | SMPD1_Ex 2.2R | GGAGCCAAATGAAGAGCACTA |
| **SMPD1 Ex3** | 368 | SMPD1_Ex 3F | TTTTACCCTCCACCCAAATG |
|  |  | SMPD1_Ex 3R | GGGAACACTTGTTGGGAATG |
| **SMPD1 Ex4** | 220 | SMPD1_Ex 4F | CAGTCCCCCTTTCTCTAGCC |
|  |  | SMPD1_Ex 4R | TGCTCAAGGGAATTTTCAGC |
| **SMPD1 Ex5** | 291 | SMPD1_Ex 5F | TGGAGAAAGAGGGCATCCTA |
|  |  | SMPD1_Ex 5R | TCCAACCTCCTTCCCCTATC |
| **SMPD1 Ex6a** | 673 | SMPD1_Ex 6.1F | GAAGGAGGTTGGAGCCAGAG |
|  |  | SMPD1_Ex 6.1R | AGCTCCAGGAAAGGAGAAGG |
| **ZEB2 Ex2** | 269 | ZEB2 Ex2F-3 | CGCGCGCGTTTCAATGGG |
|  |  | ZEB2 Ex2R-3 | CCCCCTCCTTCTCCCTGG |
| **ZEB2 Ex3** | 404 | ZEB2 Ex3F-3 | AACTAAACAATTAGGGGTGGCTG |
|  |  | ZEB2 Ex3R-3 | CTGCTAGGTGGAACAGATTAGTTG |
| **ZEB2 Ex4** | 218 | ZEB2 Ex4F-3 | CCTGTGCTTGGCATGCTTAG |
|  |  | ZEB2 Ex4R-3 | GTTTCCTTCCCTGCCTCAC |
| **ZEB2 Ex5** | 334 | ZEB2 Ex5F-3 | GATGGAGATGGACTTGGGATC |
|  |  | ZEB2 Ex5R-3 | CAGGCATGTAGTGCATTTGTAATTG |
| **ZEB2 Ex6** | 361 | ZEB2 Ex6F-3 | TCAGCCCATTAGCTGCC |
|  |  | ZEB2 Ex6R-3 | GATTGCCAATCAAAGCAATATCGTTTC |
| **ZEB2 Ex7** | 249 | ZEB2 Ex7F-3 | GTAACAAATTTTCTTTGTTCCTCCTGC |
|  |  | ZEB2 Ex7R-3 | GTGTAAATTTTAAACAATCTTTTAAAACTCCCC |
| **ZEB2 Ex8a** | 495 | ZEB2 Ex8-1F-3 | CAGATTCTTCTCTTGGTAGACAATTGC |
|  |  | ZEB2 Ex8-1R-3 | GTTCCATCCCTACACCTAAG |
| **ZEB2 Ex8b** | 477 | ZEB2 Ex8-2F-3 | GAATGGTGGGCTTGGAG |
|  |  | ZEB2 Ex8-2R-3 | TGGTTCTCAATCATTTTGTCATC |
| **ZEB2 Ex8c** | 497 | ZEB2 Ex8-3F-3 | GACTCAAGGAGACAGATCAG |
|  |  | ZEB2 Ex8-3R-3 | CTTCTTTCCAGGGATGGG |
| **ZEB2 Ex8d** | 474 | ZEB2 Ex8-4F-3 | GAAGGAATGGTTTGAACAACG |
|  |  | ZEB2 Ex8-4R-3 | TGATCTAAACTGATGCTACTAGC |
| **ZEB2 Ex8e** | 494 | ZEB2 Ex8-5F-3 | CAAAACAAATGAAAGAACCCAAAAG |
|  |  | ZEB2 Ex8-5R-3 | CCTACATGCACATAATCAAAATAATTGCC |
| **ZEB2 Ex8long** | 2,117 | ZEB2 Ex8-1F-3 | CAGATTCTTCTCTTGGTAGACAATTGC |
|  |  | ZEB2 Ex8-5R-3 | CCTACATGCACATAATCAAAATAATTGCC |
| **ZEB2 Ex9** |  | ZEB2 Ex9F-3 | CCCCCTATGTGAAGTTGTTTGG |
|  |  | ZEB2 Ex9R-3 | GAAATGTACAGCAGGACGGG |
| **ZEB2 Ex10a** | 728 | ZEB2 Ex10F-3 | GACTCGCGTACAGAATTAGTGG |
|  |  | ZEB2 Ex10R-3 | ACACAGCAGTGTTTTCAACAGG |
| **ZEB2 Ex10b** | 358 | ZEB2 Ex10F-3seq | tactctgactcggaggag |
|  |  | ZEB2 Ex10R-3 | ACACAGCAGTGTTTTCAACAGG |
| **ZEB2 Ex10c** | 388 | ZEB2 Ex10F-3 | GACTCGCGTACAGAATTAGTGG |
|  |  | ZEB2 Ex10R-3seq | ctcctccgagtcagagta |
